# Supramolecular photodynamic agents for simultaneous oxidation of NADH and generation of superoxide radical

**DOI:** 10.1038/s41467-022-33924-3

**Published:** 2022-10-19

**Authors:** Kun-Xu Teng, Li-Ya Niu, Nan Xie, Qing-Zheng Yang

**Affiliations:** 1 Key Laboratory of Radiopharmaceuticals Ministry of Education, College of Chemistry, Beijing Normal University, Beijing, 100875 P.R. China; 2 School of Pharmaceutical Sciences, Capital Medical University, Beijing, 100069 P. R. China

**Keywords:** Supramolecular polymers, Photochemistry

## Abstract

Given that Type-I photosensitizers (PSs) have hypoxia tolerance, developing general approaches to prepare Type-I PSs is of great importance, but remains a challenge. Here, we report a supramolecular strategy for the preparation of Type-I photodynamic agents, which simultaneously generate strong oxidizing cationic radicals and superoxide radicals, by introducing electron acceptors to the existing Type-II PSs. As a proof-of-concept, three electron acceptors were designed and co-assembled with a classical PS to produce quadruple hydrogen-bonded supramolecular photodynamic agents. The photo-induced electron transfer from the PS to the adjacent electron acceptor occurs efficiently, leading to the generation of a strong oxidizing PS^+•^ and an anionic radical of the acceptor, which further transfers an electron to oxygen to form O_2_^−•^. In addition, these photodynamic agents induce direct photocatalytic oxidation of NADH with a turnover frequency as high as 53.7 min^−1^, which offers an oxygen-independent mechanism to damage tumors.

## Abstract (web summary)

Tumour hypoxia is a major issue for conventional photodynamic therapies, Here, the authors report on the supramolecular assembly of electron acceptors with photosensitizers which have improved reactive oxygen species production and are able to directly oxidise NHDH and demonstrate application against hypoxic tumours.

## Introduction

Photodynamic therapy (PDT) has emerged as a promising treatment for cancer, which operates by administrating photosensitizers (PSs) to produce cytotoxic reactive oxygen species (ROS) under light irradiation^1–6^. The majority of PSs used in clinical practice or reported in literature produce singlet oxygen (^1^O_2_) through energy transfer between the PS in an excited triplet state (^3^PS*) and molecular oxygen (Type-II mechanism)^7–14^. However, the yield of ^1^O_2_ generated by Type-II PSs is highly dependent on local O_2_ concentration, meaning the Type-II PSs have a severely lower therapeutic efficacy in treating hypoxic solid tumors, which restricts its application^15–19^. In contrast, Type-I PSs have been reported to have the benefit of less oxygen content dependency, though this mechanism is not yet well understood^20–27^. They produce cytotoxic radicals by electron transfer among ^3^PS*, adjacent substrates, and molecular oxygen^28–32^.

**Fig. 1:**
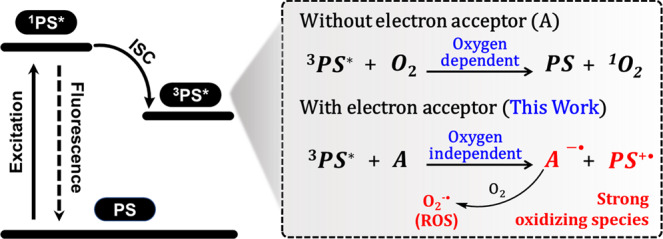
Mechanism for the generation of oxidizing species. The relax pathways of ^3^PS* with or without electron acceptor.

Unfortunately, Type-I PSs are much less reported, compared to Type-II PSs, due to the lack of general molecular design approaches^33–35^. This situation is created mainly because the electron transfer reaction is always too inefficient to compete with the energy transfer of the ^3^PS* to molecular oxygen^36,37^. It remains a challenge to control the relative contributions of energy transfer and electron transfer by molecular design^38,39^. In the reported Type-I PDT reactions, the ^3^PS* first abstracts an electron from nearby substrates, generating an anion radical (PS^−•^), which further transfers an electron to molecular oxygen to produce a superoxide radical (O_2_^−•^)^40,41^. This is the widely accepted Type-I mechanism, but is normally inefficient because PSs cannot effectively collide with nearby substrates^42,43^. Theoretically, ^3^PS* also can transfer an electron to an electron-deficient substrate, and then to O_2_ to produce O_2_^−•^^44,45^. However, such a Type-I PDT mechanism has never been reported in the literature, presumably due to the lack of suitable electron-deficient substrates in biological systems^46^. We anticipate that introducing appropriate electron acceptors close to the existing Type-II PS would facilitate the occurrence of an efficient electron transfer between PS and acceptors, leading to the generation of ROS by the Type-I reaction (Fig. 1). This strategy has never been proposed and demonstrated in the literature. It has the following two advantages: (i) it can be employed to convert the existing Type-II photosensitizers to Type-I photosensitizers; (ii) it not only promotes the generation of O_2_^−•^, but also yields a strong oxidizing cationic radical of the photosensitizer (PS^+•^) that directly oxidizes biomolecules (e.g., NADH) to cause biological damage in an oxygen-independent manner.

**Fig. 2:**
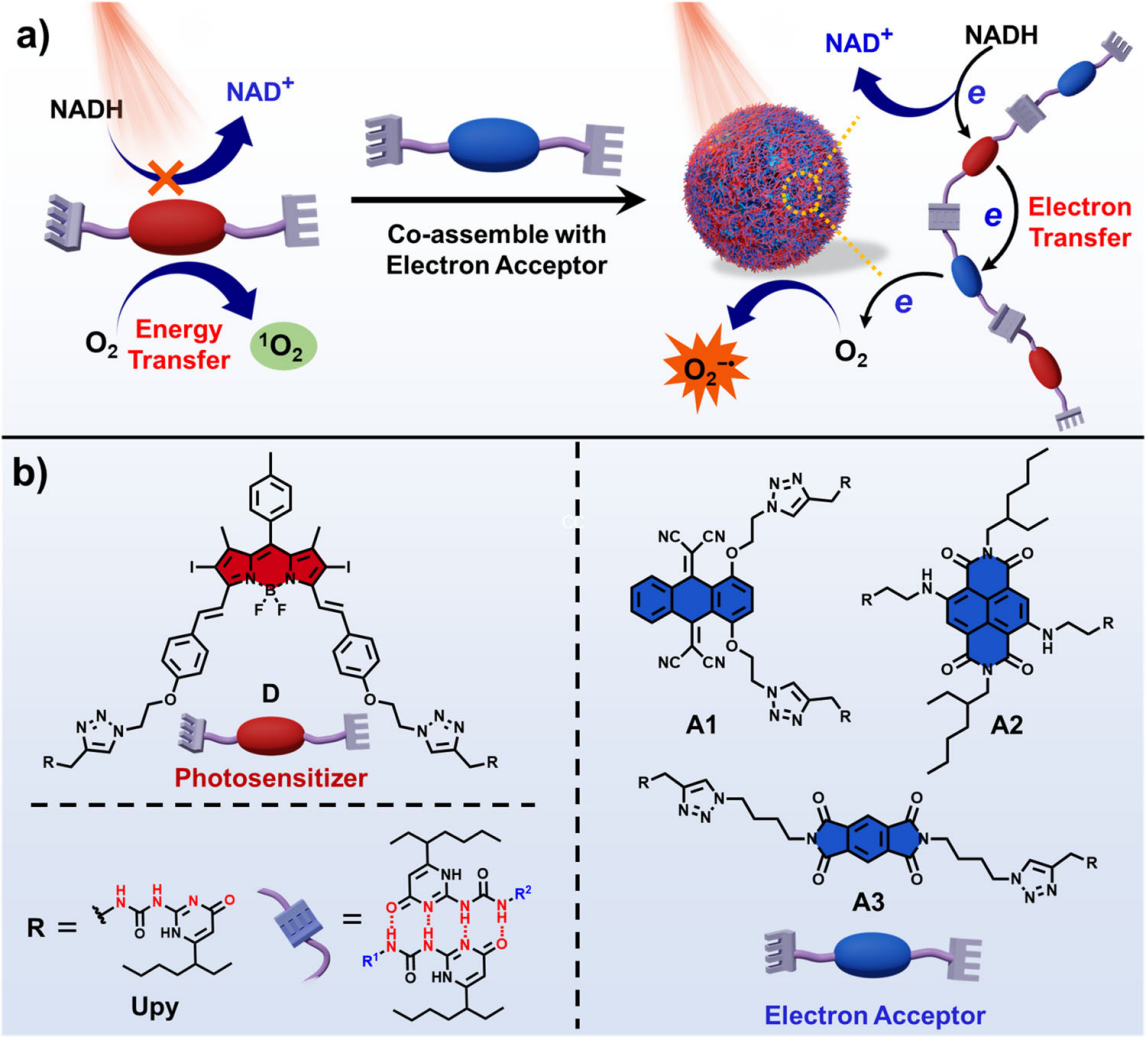
Schematic representation of PDT based on supramolecular photodynamic agents. **a** Schematic illustration of the preparation of the supramolecular photodynamic agent, as well as photo-induced oxidation of NADH and generation of O_2_^−•^. **b** Chemical structures of the photosensitizer (**D**) and electron acceptors (**A1**, **A2**, and **A3**).

Here, we show a powerful strategy for enhancing the Type-I PDT reactions by introducing electron acceptors to the existing Type-II PS through the supramolecular assembly. As a proof-of-concept, three different types of electron acceptors are designed and co-assembled with a conventional Type-II PS to form quadruple hydrogen-bonded supramolecular photodynamic agents (Fig. 2). Proximity of the PS to electron acceptors with matched redox potential promotes, upon light irradiation, the electron transfer from the PS to the electron acceptor, then to O_2_, which leads to an efficient generation of O_2_^−•^. In addition, this process is accompanied by the generation of a strong oxidizing cationic radical of the PS (PS^+•^), which effectively oxidizes 1,4-dihydronicotinamide adenine dinucleotide (NADH), an important coenzyme in living cells, aggravating biological damage of tumor cells in an oxygen-independent manner. Strikingly, these supramolecular PSs show the highest NADH oxidation turnover frequency (TOF) of 53.7 min^−1^, which is more than 1–3 orders of magnitude higher than those of conventional transition-metal catalysts. As a result, the supramolecular PS exhibited superior PDT performance in treating tumor cells even under hypoxic environments (2% O_2_). Distinct solid tumor ablation in vivo was also achieved in the tumor treatment of mouse models.

## Results and discussion

A classical Type-II PS, iodide-BODIPY (**D**)^47,48^, and three different types of electron-deficient acceptors (**A1**, **A2**, **A3**) were modified with quadruple hydrogen-bonding units 2-ureido-4[1H]-pyrimidinone (UPy)^49^, which is one of the most commonly used building blocks in supramolecular assembly (Fig. 2b)^50,51^. The synthetic routes are illustrated in the Supplementary Information. All products were characterized by ^1^H NMR, ^13^C NMR, and HR-MS.

**Fig. 3:**
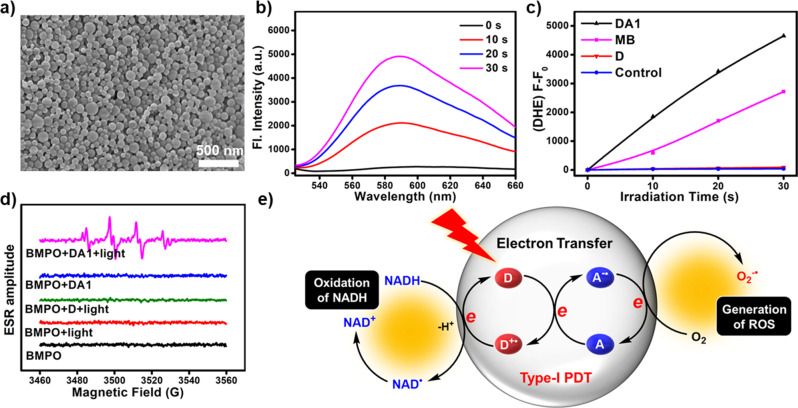
Morphology and ROS generation properties of DA1. **a** SEM image of DA1. The experiment was repeated three times independently, with similar results. Scale bar: 500 nm. **b** The fluorescence spectra of DHE (40 μM, excitation at 510 nm, detection from 525 to 660 nm) containing 500 μg mL^−1^ ctDNA after irradiation (660 nm, 20 mW cm^−2^) for a different time in the presence of **DA1**. **c** Plots of ΔFl. (F-F_0_) of DHE at 580 nm upon light irradiation (660 nm, 20 mW cm^−2^) for different time intervals in the presence of **DA1**, MB, or **D**. **d** ESR spectra to detect O_2_^−•^ generated by **DA1** (0.5 mM) under illumination, using BMPO (25 mM) as a spin-trap agent. **e** Schematic illustration of the photoinduced oxidation of NADH and generation of O_2_^−•^.

The electron acceptor of **A1** was first chosen to assemble with **D** to form photodynamic agent **DA1** (Supplementary Fig. 1). The formation of the supramolecular polymer was confirmed by diffusion-ordered NMR spectroscopy (DOSY). The diffusion coefficient decreased from 6.31 × 10^−10^ to 1.32 × 10^−10^ m^2^ s^−1^ as the mixture of **D** and **A1** (molar ratio 4:6) increased from 10 to 50 mM, suggesting the formation of supramolecular polymers (Supplementary Fig. 2). Scanning electron microscopy (SEM) image revealed that **DA1** were uniformly distributed in a spherical shape and the average hydrodynamic diameter of **DA1** estimated by dynamic light scattering was 69.2 ± 6.4 nm (Fig. 3a and Supplementary Fig. 3). The quadruple hydrogen-bonds are crucial in assembly and formation of nanoparticles with well-defined size and morphology (Supplementary Fig. 4). The aqueous dispersion of **DA1** exhibits excellent photostability and thermal stability in PBS and complete medium, which is beneficial to the biological application (Supplementary Fig. 5). The aqueous dispersion of **DA1** absorbs at 665 nm and emits weak fluorescence at 730 nm (Supplementary Fig. 6).

The enhancement of the Type-I PDT reactions for **DA1** was initially verified by evaluating the generation of O_2_^−•^ with the specific O_2_^−•^ indicator dihydroethidium (DHE). Upon light irradiation, the fluorescence of the DHE solution in the presence of **DA1** increases significantly, indicating that **DA1** generates O_2_^−•^ efficiently (Fig. 3b). The increase of fluorescence intensity for **D** is negligible, suggesting that no O_2_^−•^ is generated without electron acceptor (Supplementary Fig. 7). As shown in Fig. 3c, the rate of O_2_^−•^ generated by the irradiation of **DA1** is 1.7-fold that of methylene blue (MB) under the identical condition. Then, 9,10-anthracenediyl-bis(methylene)dimalonic acid (ABDA) was employed as a ^1^O_2_ indicator to evaluate the ^1^O_2_ generation of **DA1**. The ^1^O_2_ generation rate of **DA1** is markedly reduced compared with **D** (Supplementary Fig. 8), suggesting the Type-II PDT process of energy transfer is suppressed due to the conversion to the electron transfer. In addition, electron spin resonance (ESR) spectroscopy was employed to confirm the generation of O_2_^−•^ (Fig. 3d). Upon light irradiation of **DA1** in aqueous solution with BMPO as a spin-trap agent for O_2_^−•^, a characteristic signal of the adduct of O_2_^−•^ with BMPO was observed, further confirming the generation of O_2_^−•^. The above results demonstrate that introducing an electron acceptor to the Type-II PS is an effective strategy for converting the Type-II process to Type-I.

Then, we propose the mechanism of the photoinduced generation of O_2_^−•^ (Fig. 3e). The triplet excited photosensitizer (^3^D*) formed by photoirradiation transfers an electron to a nearby electron acceptor (**A**) to form radical ions **D**^+•^ and **A**^−•^. The anionic radical **A**^−•^ further transfers the electron to molecular oxygen to produce O_2_^−•^. Notably, the cationic radical **D**^+•^ is a strong oxidizing agent, which can directly oxidize biomolecules to cause biological damage in an oxygen-independent manner.

**Fig. 4:**
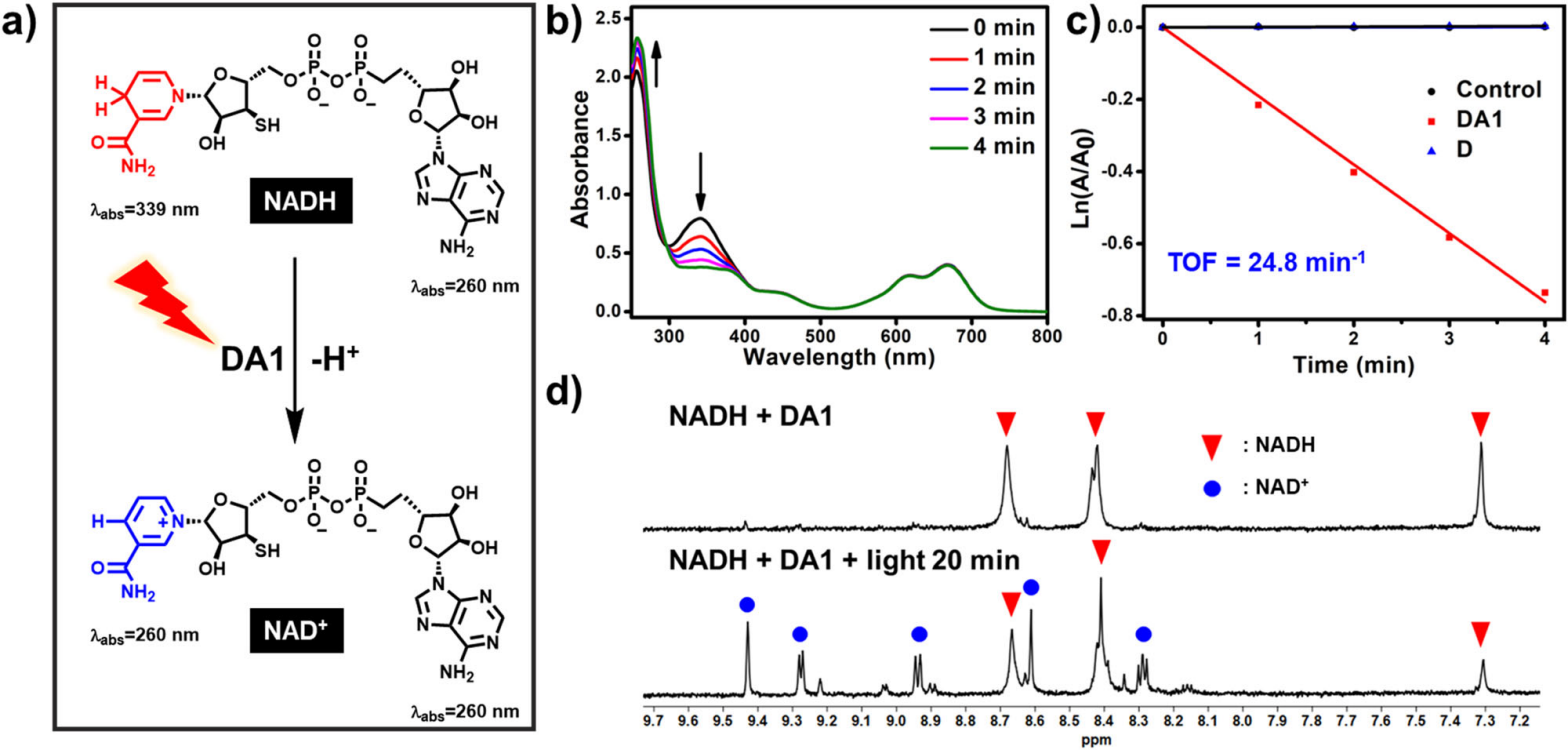
Oxidation of NADH. **a** Schematic illustration of the photocatalytic NADH/NAD^+^ transformation. **b** The oxidation of NADH (100 μM) by **DA1** (10 μM) upon irradiation (660 nm, 20 mW cm^−2^) in PBS solution, as monitored by ultraviolet-visible spectroscopy. **c** Plots of lnA/A_0_ of NADH at 339 nm for different time intervals. **d** ^1^H NMR of NADH in D_2_O containing **DA1**.

According to the above mechanism, the photo-oxidation of NADH by **DA1** was evaluated. NADH, as an important coenzyme in many oxidoreductases, participates in the maintenance of the intracellular redox balance^52–54^. The oxidization of NADH in cancer cells would disturb the intracellular redox balance, thus leading to cell death^55–57^. When NADH is oxidized to NAD^+^, the absorption at 339 nm is reduced and the absorption at 260 nm is enhanced (Fig. 4a). As shown in Fig. 4b, c, upon light irradiation the solution of NADH in the presence of **DA1**, the absorption of NADH at 339 nm decreases significantly, whereas it keeps stable when only NADH is present. This result indicates that NADH is photo-oxidized and catalyzed by **DA1**. Excitingly, the NADH oxidation TOF was calculated to be 24.8 min^−1^, which is much more efficient than widely explored transition-metal-containing photocatalysts^53,57^. The decrease of NADH absorption is negligible in the presence of **D** after irradiation for 4 min, suggesting that it cannot be efficiently oxidized by ^1^O_2_ (Supplementary Fig. 9). To further confirm the photo-oxidation of NADH in the presence of **DA1**, ^1^H NMR spectroscopy was employed to monitor the conversion from NADH to NAD^+^ (Fig. 4d and Supplementary Fig. 10). The newly formed peaks of NAD^+^ were observed at 8.29, 8.62, 8.94, 9.28, and 9.44 ppm in the presence of **DA1**, confirming the photo-oxidative reaction from NADH to NAD^+^ upon the light irradiation^53^. In the absence of **DA1**, no NAD^+^ was detected.

**Fig. 5:**
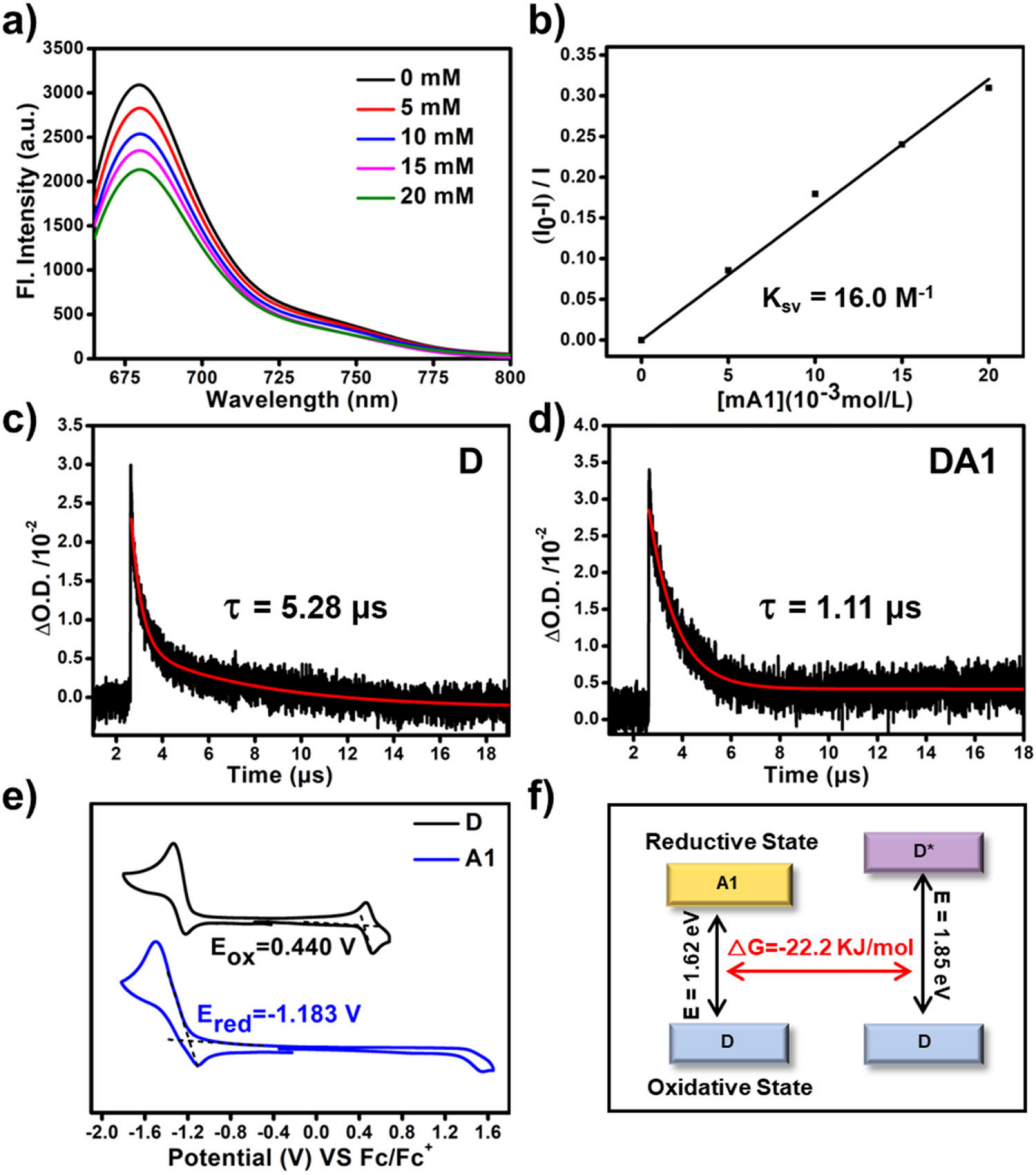
The study of electron transfer. **a** The emission spectra of **D** (1.0 × 10^−5^ M) at 25 °C in DMF with increasing amounts of **A1** (0–20 mM) under excitation at 650 nm. **b** The Stern–Volmer plots for the fluorescence quenching of **D** by **A1** in DMF (I_0_ is the fluorescence intensity of **D** in the absence of **A1** and I is the fluorescence intensity in the presence of **A1** at the different concentrations). Nanosecond transient absorption spectra: Decay trace of **D** in the **c** supramolecular polymer without electron acceptor and **d** **DA1** dispersed in water (20 μM); the excitation wavelength was 660 nm and the monitoring wavelength was 550 nm. Cyclic voltammogram of **e** **D** and **A1** in DCM with 0.1 M (n-Bu)_4_N^+^PF_6_^-^ as a supporting electrolyte, Ag/Ag^+^ as a reference electrode, glassy-carbon electrode as a working electrode, and Pt wire as a counter electrode; scan rate, 100 mV s^−1^; Fc/Fc^+^ was used as an external reference. **f** Gibbs free energy of electron transfer between **D** and **A1**.

In order to understand the formation of O_2_^−•^ and the oxidation of NADH, the electron transfer from exited **D** to **A1** was studied. The Stern–Volmer quenching of the emission of **D** with **A1** was initially performed. As shown in Fig. 5a, the emission of **D** is gradually quenched with the addition of **A1**. The Stern–Volmer plots show a linear correlation between the concentration of **A1** and the (I_0_-I)/I ratio, suggesting that **A1** quenches the excited state of **D** by electron transfer (Fig. 5b). In addition, the lifetime of the triplet state of **D** in the supramolecular agent **DA1** was measured by laser flash photolysis (Supplementary Fig. 11). As shown in Fig. 5c, d, the lifetime of the triplet state of **D** in the **DA1** compared with that of **D** without electron acceptor of **A1** decreases from 5.28 to 1.11 μs, indicating that **A1** quenched the excited triplet state of **D** by electron transfer. In order to further study the intermolecular electron transfer, cyclic voltammetry was conducted to determine the oxidation potential (E^ox^) of **D** and the reduction potential (E^red^) of **A1**. As shown in Fig. 5e, the E^ox^ of **D** is +0.440 V (VS Fc/Fc^+^) and the E^red^ of **A1** is −1.183 V (VS Fc/Fc^+^). According to the Rehm–Weller equation, the Gibbs free energy (ΔG) of electron transfer between **D** and **A1** was estimated as −22.2 kJ mol^−1^, confirming the thermodynamic feasibility of intermolecular electron transfer (Fig. 5f and Supplementary Fig. 12).

To verify the universality of this strategy, we further evaluated the photoinduced O_2_^−•^ generation and NADH oxidation of **DA2** and **DA3**. As shown in Supplementary Fig. 13, the rate of O_2_^−•^ generated by the irradiation of **DA2** and **DA3** is 2.0- and 1.6-fold that of MB under the identical condition and the total ROS generation efficiencies of supramolecular photodynamic agents are enhanced compared to their monomers (Supplementary Fig. 14). The ESR spectroscopy was further employed to confirm the O_2_^−•^ generation by sensitization of **DA2** and **DA3** (Supplementary Fig. 15). Gratifyingly, **DA2** and **DA3** also efficiently oxidize NADH with TOF of 53.7 and 24.3 min^−1^, respectively (Supplementary Fig. 16). Then, the Stern–Volmer quenching titration of the emission of **D** was employed with **A2** and **A3**, and the Stern–Volmer quenching constants, *K*_sv_, are 7.7 and 4.9 M^−1^, respectively (Supplementary Fig. 17). In addition, the calculated ΔG of electron transfer between **D** and **A2** or **A3** are both negative and the lifetime of a triplet of **D** is also decreased in the presence of **A2** or **A3** (Table 1 and Supplementary Figs. 18, 19). All the above results show that the introduction of an electron acceptor enhances the electron transfer from the PS to the electron acceptor, thereby generating O_2_^−•^ and oxidizing NADH.

**Table 1:** The relevant data on photoinduced electron transfer between **D** and **A**

Electron acceptor	*K*_sv_ (M^−1^)^a^	E^red^ (V)^b^	ΔG (KJ mol^−1^)^c^	τ_T_ (μs)^d^
**A1**	16.0	−1.183	−22.2	1.11
**A2**	7.7	−1.106	−29.9	0.96
**A3**	4.9	−1.175	−23.2	2.17

^a^The Stern–Volmer quenching constants (*K*_sv_) were measured by titration.

^b^Reduction potentials were measured by cyclic voltammetry with ferrocene as the standard.

^c^The Gibbs free energy (ΔG) was calculated according to the Rehm–Weller equation.

^d^The lifetime of the triplet state (τT) of **D** in the supramolecular agent was measured by laser flash photolysis.

**Fig. 6:**
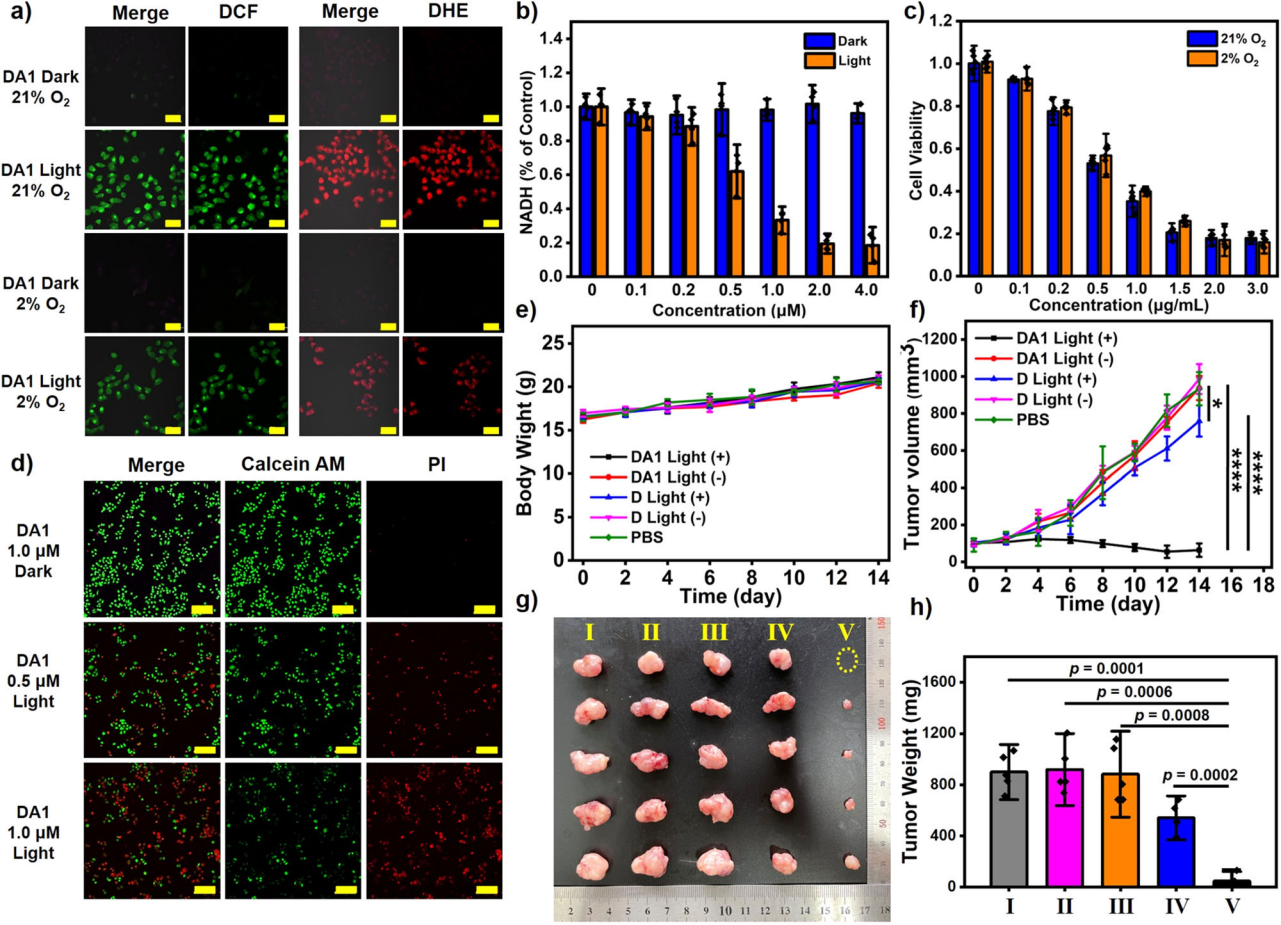
PDT efficacy of supramolecular photosensitizers in vitro and in vivo. **a** Detection of ROS and O_2_^−•^ in HeLa cells with DCFH-DA and DHE under normoxic (21% O_2_) or hypoxic (2% O_2_) conditions. The scale bar represents 50 μm. **b** Quantitative analysis of intracellular NADH level in Hela cells subjected to a range of **DA1** concentrations. Data were presented as mean ± s.d. derived from *n* = 5 independent biological samples. **c** Cell viability of HeLa cells subjected to a range of **DA1** concentrations in the presence of light irradiation under normoxic or hypoxic conditions. Data were presented as mean ± s.d. derived from *n* = 6 independent biological samples. **d** CLSM images of calcein-AM/PI-stained HeLa cells. The scale bar represents 200 μm. **e** Body weight of the mice during the observation. **f** Tumor growth profiles during the observation. ****p < 0.0001, *p = 0.012. **g** Images of tumor tissues from different groups of tumor-bearing mice. **h** Average tumor weight of different groups of tumor-bearing mice. I: PBS; II: **D** Light (−); III: **DA1** Light (−); IV: **D** Light (+); V: **DA1** Light (+). Data in (**e**, **f**, **h**) are presented as mean ± s.d. derived from *n* = 5 independent biological samples. Statistical significance was assessed via an unpaired two-sided student *t*-test.

Taking **DA1** as an example, the PDT activity was evaluated in vitro. **DA1** is internalized by HeLa cells and reaches the maximum at about 6 h after incubation (Supplementary Fig. 20). ROS indicator 2′,7′-dichlorodihydrofluorescein diacetate (DCFH-DA) and a specific O_2_^−•^ probe DHE, were used to detect the cellular ROS generation during PDT in normoxic (21% O_2_) and hypoxic (2% O_2_) environment. As shown in Fig. 6a, after illumination, HeLa cells treated with **DA1** and DCFH-DA or DHE manifest obvious fluorescence signals, indicating that **DA1** produces ROS efficiently under either normoxic or hypoxic conditions. Then we investigated NADH depletion in HeLa cells with NAD^+^/NADH assay kit with WST-8. As shown in Fig. 6b, the intracellular NADH levels reduced with the increment of **DA1** concentration upon light irradiation, while in the absence of light irradiation, the NADH levels were unaffected. The PDT effect of **DA1** on HeLa cells was further evaluated by cell counting kit-8 (CCK-8) assays. As shown in Supplementary Fig. 21, there is no toxicity of **DA1** to HeLa cells without irradiation in both normoxic and hypoxic environments. After irradiation with 660-nm LED light (20 mW cm^−2^) for 10 min, **DA1** showed obvious cytotoxicity to HeLa cells with a half-maximal inhibitory concentration (IC_50_) of 0.88 μM under normoxia and 1.15 μM under hypoxia (Fig. 6c). Then, Calcein-AM and propidium iodide (PI) assays were performed to further investigate the apoptosis process of HeLa cells during PDT, in which viable cells were stained by calcein-AM to emit fluorescence in the green channel and apoptotic cells were stained by PI to emit fluorescence in the red channel. As shown in Fig. 6d, HeLa cells treated with **DA1** and illumination emit fluorescence in both green and red channels, indicating partial cell death. The proportion of red fluorescence increased with the increase of **DA1** concentration, suggesting the inhibition of HeLa cells by **DA1** under irradiation. By contrast, HeLa cells without illumination merely exhibited fluorescence in the green channel under identical conditions, indicating no cell death.

Motivated by the excellent experiment results in vitro, the PDT efficiency of **DA1** was further investigated in immunodeficient mouse models by using a subcutaneous tumor model of HeLa in the BALB/c mice. The fluorescence imaging in mice indicated **DA1** was enriched in tumor position due to the enhanced permeability and retention (EPR) effect (Supplementary Fig. 22). Then we proceeded to study the antitumor efficiency of **DA1** in mice bearing HeLa tumor (primary tumor volume: ~100 mm^3^). **DA1** (130 μM, 60 μL) was subjected to the mice by tail vein injection, followed by illumination treatment (660 nm, 120 mW cm^−2^) on tumor sites at 9 h post intravenous injection. The body weights (Fig. 6e) and tumor volumes (Fig. 6f) were recorded during the subsequent 14 days. The weight of the mice increased slightly, indicating that the systemic cytotoxicity of **DA1** is negligible. For the group treated with **DA1** and light, the growth of tumor was inhibited compared with the control group, suggesting that **DA1** effectively suppressed the tumor in vivo. And the anti-tumor effect of **D** without an electron acceptor under identical condition was obviously inferior to that of **DA1**, suggesting that the introduction of electron acceptors can significantly improve the efficacy of PDT. The mice were sacrificed on the 14th day and all tumor tissues were peeled and weighed. As shown in Fig. 6g, h, the volume and weight of tumor of the treatment group were significantly smaller than that of the control groups, indicating that **DA1** well inhibits tumors in vivo.

In conclusion, we report the preparation of Type-I photodynamic agents by introducing electron acceptors to existing Type-II PSs through the quadruple hydrogen-bonded supramolecular assembly. The photoinduced electron transfer from the PS to the electron acceptor is realized by the shortened distance between the PS and the electron acceptor with matched redox potential. Such a process enhances the formation of radical ion pairs. On the one hand, the anionic radical of the acceptor transfers an electron to molecular oxygen to produce O_2_^−•^; on the other hand, the cationic radical of the photosensitizer efficiently oxidizes NADH to cause biological damage with an oxygen-independent mechanism, enhancing the PDT efficacy. Experiments show that the prepared photodynamic agents efficiently produce O_2_^−•^ and oxidize NADH with a TOF as high as 53.7 min^−1^ upon light irradiation, and they exhibit excellent PDT performance, even under a hypoxic environment. We envision that this work will broaden the insight into Type-I PDT and inspire researchers to develop effective photodynamic agents for cancer treatment.

## Methods

All reagents and solvents were obtained from commercial suppliers and were used without further purification. Cell culture media, PBS, fetal calf serum (FBS), and CCK-8 (cell counting kit-8) were purchased from Yeasen Biotechnology (Shanghai) Co., Ltd. DCFH-DA (2’,7’-dichlorodihydrofluorescein diacetate), DHE (Dihydroethidium), WST-8 (NAD^+^/NADH Assay Kit), and Calcein-AM/propidium iodide (PI) detection Kit were purchased from Beyotime Biotechnology (Shanghai) Co., Ltd. All chemical synthesis reagents are purchased from Energy Chemical (Shanghai) Co., Ltd. and TCI Chemical Co., Ltd. Female BALB/c mice (6–8 weeks) were used as an animal model in this study. All mouse models are provided by Beijing Vital River Laboratory Animal Technology Co., Ltd. Mice were housed in individually ventilated cage (IVC) systems (ambient temperature: 23 ± 3 °C; relative humidity: 40–70%). The instruments and equipment involved in the article are listed in Supplementary Information.

### Data analysis

All plotted and calculated statistical analyses were performed on Origin 8.5 and SPSS.

### Ethical statement

All animal experiments were performed following the protocols evaluated and approved by the Animal Ethics Committee of Capital Medical University (Ethics Approval Number: AEEI-2018-097).

### In vitro O_2_^−•^ detection by DHE

DHE can be oxidized by O_2_^−•^ to produce ethidium which intercalates into RNA/DNA and emits fluorescence at 580 nm. About 10 µM of PSs was dispersed in 2 mL PBS containing 40 µM of DHE and 500 μg mL^−1^ ctDNA. Upon the irradiation (660 nm LED light, 20 mW cm^−2^), the fluorescence intensity of DHE at 580 nm was recorded by the fluorescence spectrometer.

### In vitro ^1^O_2_ detection by ABDA

9,10-anthracenediyl-bis(methylene)-dimalonic acid (ABDA) can be oxidized by ^1^O_2_ and the absorption at 378 nm is reduced. About 10 µM of PSs and 30 µM of ABDA were dispersed in 2 mL PBS. Upon the irradiation (660 nm LED light, 20 mW cm^−2^), the absorption intensity of ABDA at 378 nm was recorded by the UV-Vis absorption spectrometer.

### In vitro O_2_^−•^ detection by ESR spectroscopy

BMPO (5-*tert*-butoxycarbonyl 5-methyl-1-pyrroline *N*-oxide) was employed as a spin-trap agent for O_2_^−•^ in vitro. ESR spectroscopy was employed to detect the ESR signals of the following five groups of samples: (i) 100 µM of **DA1** was dispersed in water containing 25 mM of BMPO and illuminated with a xenon lamp (500–1200 nm); (ii) 100 µM of **DA1** was dispersed in water containing 25 mM of BMPO without light; (iii) 100 µM of **D** was dissolved in DMSO containing 25 mM of BMPO and illuminated with a xenon lamp; (iv) 25 mM of BMPO was dissolved in DMSO and illuminated with a xenon lamp; (v) 25 mM of BMPO was dispersed in water without light.

### Calculation of turnover frequency (TOF)

The TOF of photocatalytic oxidation of NADH was calculated from the difference in NADH concentration after 1 min irradiation divided by the concentration of photosensitizer. The concentration of NADH was calculated using the extinction coefficient ε_339_ = 6220 M^−1^cm^−1^ in PBS buffer.

### Cyclic voltammetry measurement

We employed a three-electrode system to conduct the cyclic voltammograms experiment. Specifically, a platinum-carbon compound electrode was used as the working electrode, the Pt wire electrode was used as the auxiliary electrode, and the Ag/Ag^+^ electrode was used as the reference electrode. The measurement was conducted in dichloromethane and 0.1 M tetrabutylammonium hexafluorophosphate was added as a supporting electrolyte. The scan rate was 100 mV/s. Fc/Fc^+^ was used as an external reference.

### ROS detection in living cells

DCFH-DA was used as a ROS indicator to detect intracellular ROS generation. Human cervical cancer (HeLa) cells were planted onto 35-mm confocal dishes at a density of 1 × 10^5^ cells. After incubation for 24 h under normoxic (21% O_2_) or hypoxic (2% O_2_) conditions, the medium was removed and washed with PBS three times. Fresh medium containing 0.5 μM **DA1** was added and incubated for another 6 h. Then, the medium was replaced with fresh medium containing 2 μM DCFH-DA and incubated for 0.5 h. After that, the medium was removed and washed with PBS three times. After irradiation with a 660 nm LED light (40 mW cm^−2^) for 5 min, fluorescence images were promptly captured by confocal laser scanning microscopy (CLSM).

### O_2_^−•^ detection in living cells

DHE was used as an O_2_^−•^ indicator to detect intracellular O_2_^−•^ generation. HeLa cells were planted onto 35-mm confocal dishes at a density of 1 × 10^5^ cells. After incubation for 24 h under normoxic (21% O_2_) or hypoxic (2% O_2_) conditions, the medium was removed and washed with PBS three times. Fresh medium containing 0.5 μM **DA1** was added and incubated for another 6 h. Then, the medium was replaced with fresh medium containing 2 μM DHE and incubated for 0.5 h. After that, the medium was removed and washed with PBS three times. After irradiation with a 660 nm LED light (40 mW cm^−2^) for 5 min, fluorescence images were promptly captured by the CLSM.

### Intracellular NADH determination

Experiments to determine the NADH changes in Hela cells treated with different concentrations of **DA1** were performed using the NAD^+^/NADH assay kit with WST-8. In brief, Hela cells were seeded in six-well plates and incubated for 8 h. Then, the HeLa cells were exposed to different concentration of **DA1**, i.e., 0, 0.1, 0.2, 0.5, 1.0, 2.0, and 4.0 μM. After incubation for another 6 h, the cells were washed with PBS buffer three times, infused with fresh DMEM, and illuminated by a 660 nm LED light (20 mW cm^−2^) for 10 min. After further incubation for 8 h, the NADH concentration was examined with an NAD^+^/NADH assay kit. For NAD^+^/NADH extraction, 200 μL of NAD^+^/NADH extraction buffer was added and blow gently, followed by spinning at 10,000 rpm (5 min, 4 °C) to get the supernatant as a test sample. After heating the collected supernatant at 60 °C for 0.5 h in a water bath, cellular NADH content was finally quantified according to the instructions.

### In vitro cytotoxicity

HeLa cells were seeded onto 96-well plates at a density of 5 × 10^3^ cells and incubated for 24 h under normoxic (21% O_2_) and hypoxic (2% O_2_) conditions, respectively. A fresh DMEM medium containing various concentrations of **DA1** was added to replace the previous medium. After incubation for another 6 h, replace the medium again and illuminate with a 660 nm LED light (50 mW cm^−2^) for 10 min. the cell viability was examined by CCK-8 assays after 12 h. In addition, the dark toxicity of **DA1** was also detected by the above procedure.

### Live/dead cell staining assay

Calcein-AM and PI were used to stain the live/dead cell. HeLa cells were seeded on 35 mm confocal dishes at a density of 1 × 10^5^ cells and incubated for 24 h. Fresh medium containing various concentrations of **DA1** was added to the dishes. After incubation for 6 h, 5 μM Calcein-AM and 5 μM PI were added and incubated for another 20 min. Then, the medium was removed and washed with PBS three times. After irradiation with a 660 nm LED light (40 mW cm^−2^) for 5 min, fluorescence images were promptly captured by confocal laser scanning microscopy (CLSM).

### In vivo imaging

Tumor imaging in vivo were studied with a subcutaneous HeLa tumor model in the immunocompetent BALB/c mice. **DA1** (400 μg mL^−1^, 60 μL) were subjected to the mice by tail vein injection, followed by fluorescence imaging recorded at 1, 3, 6, and 12 h by an animal imaging system.

### Ex vivo biodistribution of DA1

After 12 h of tail vein injection, mice were sacrificed and their major organs, including heart, liver, spleen, lung, kidney, pancreas, and tumor were carefully removed for visualization under the imaging system.

### In vivo PDT experiment

The HeLa tumor-bearing mice were divided into five groups (each group contains five mice): Group I: PBS injection; Group II: **D** injection without irradiation; Group III: **DA1** injection without irradiation; Group IV: **D** injection with irradiation; Group V: **DA1** injection with irradiation. After 9 h of tail vein injection, a 660 nm LED light (120 mW cm^−2^) treatment was performed on Group IV and Group V by irradiating the tumor region for 20 min. The effect of the different treatment groups was monitored by measuring tumor size (tumor size = width^2^ × length/2) and mice body weight for 14 days after PDT treatment. After 14 days, the tumors of the mice were dissected and weighed.

### Reporting summary

Further information on research design is available in the Nature Research Reporting Summary linked to this article.

## Supplementary information

Supplementary Information

Reporting Summary

## Data Availability

The data generated in this study are available within the article, Supplementary Information, and Source Data.  Source data are provided with this paper.

## Source data

Source Data
