# Supplementary material for: Supramolecular photodynamic agents for simultaneous oxidation of NADH and generation of superoxide radical
Source: Nat Commun. 2022 Oct 19;13:6179. doi: 10.1038/s41467-022-33924-3 (PMC9582220; doi:10.1038/s41467-022-33924-3)
Supplement: Supplementary file 1 — Supplementary Information [file 41467_2022_33924_MOESM1_ESM.pdf]

Supplementary Information

Supramolecular Photodynamic Agents for Simultaneous Oxidation of NADH and Generation of Superoxide Radical

Kun-Xu Teng[1], Li-Ya Niu[1], Nan Xie[2] and Qing-Zheng Yang[1,*]

[1]Key Laboratory of Radiopharmaceuticals, Ministry of Education, College of Chemistry, Beijing Normal University, Beijing 100875, P. R. China.

[2]School of Pharmaceutical Sciences, Capital Medical University, Beijing 100069, P. R. China;

Email: qzyang@bnu.edu.cn

Table of Contents

1. General information 2
2. Synthesis of photosensitizer and electron acceptors. 3
3. The preparation and properties of supramolecular PS. 8
4. The evaluation of ROS generation ability. 13
7. Experimental data in vitro and in vivo 23
8. NMR spectra and HRMS 25
9. References 34

1. General information

All reactions were performed in dry glassware. 200-300 mesh silica gel was used to column chromatography purification. ^{1}H- and ^{13}C-NMR spectra were recorded with JEOL-400, JEOL-600 spectrometers at 298 K. DOSY spectra were recorded with Bruker Avance Drx 500 spectrometer at 298 K. High-resolution mass spectrometry (HRMS) experiments were recorded by an Agilent Technologies 6224 Accurate-Mass time-of-flight spectrometer. Absorption spectra of liquid samples were determined on Hitachi UV-3900 spectrophotometer at room temperature. Fluorescence spectra of liquid samples were determined on Hitachi F-4600 spectrophotometer at room temperature. Cyclic voltammetry was carried out with CHI760E electrochemical workstation. Dynamic light scattering (DLS) investigations were recorded with a DynaPro NanoStar dynamic light scattering detector. Scanning electron microscope (SEM) images were obtained using a Hitachi SU-8010 instrument. The photostability was conducted under irradiation with a high-power LED light and monitored by using an UV-3900 spectrophotometer. Electron spin resonance was performed with Bruker E500. Confocal fluorescence imaging was performed with Nikon A1R microscopy. Cell viability test was obtained on a Thermo Scientific Multiskan. Irradiation was performed by using a LED light (660 nm, PLS-LED 100C, Beijing Perfectlight Technology Co., Ltd). In vivo imaging was recorded by an IVIS Spectrum imaging system (PerkinElmer, USA).

2. Synthesis of photosensitizer and electron acceptors.

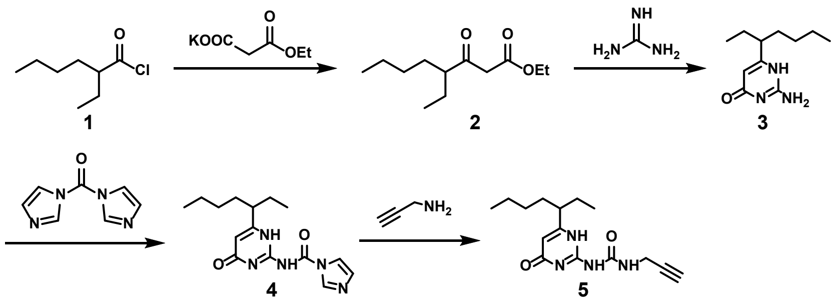

Supplementary Scheme 1 | Synthesis of supramolecular assemble units of quadruple hydrogen bonds.

Synthesis of compound **4** is referred to previous literature.[1]

Synthesis of compound **5**: Compound **4** (3.03 g, 10.0 mmol) and mono-Propargylamine (0.66 g, 12.0 mmol) were mixed in 10 mL anhydrous dichloromethane and the resulting mixture was stirred at room temperature for 8 hours under N_2 atmosphere. After the reaction was completed, the resulting mixture was washed with 2M HCl, saturated aqueous $NaHCO_3$, brine and dried over anhydrous Na_2SO_4. Evaporation of the solvent under reduced pressure and the further purification was carried out by column chromatography using CH_2Cl_2/CH_3OH (100:1, v/v) as eluent to afford the product as colorless oil (2.73 g, 94 %). ^1H NMR (600 MHz, Chloroform-*d*) δ 13.01 (s, 1H), 12.11 (s, 1H), 10.83 (s, 0H), 5.85 (s, 1H), 4.06 (dd, *J* = 5.2, 2.5 Hz, 2H), 2.31 (ddd, *J* = 14.7, 9.2, 5.5 Hz, 1H), 2.25 (t, *J* = 2.5 Hz, 1H), 1.76 – 1.46 (m, 4H), 1.38 – 1.10 (m, 4H), 0.88 (m, 5H).

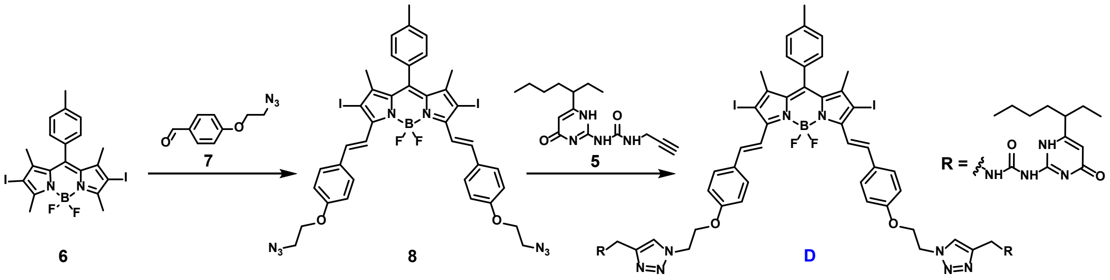

Supplementary Scheme 2 | Synthesis of compound D.

Synthesis of compound **6** is referred to previous literature.[2]

Synthesis of compound **8**: Compound **6** (590.0 mg, 1.0 mmol) and compound **7** (448.0 mg, 2.5 mmol) were added into a two-neck vial. The Schlenk tube was capped with a rubber septum and then evacuated and backfilled with N_2 (this sequence was carried out three times). Glacial acetic acid (0.6 mL), piperidine (0.8 mL) and toluene (20 mL) were added via syringe through the septum, and the Schlenk tube was sealed. The reaction mixture was heated to 100 °C until **6** had been completely consumed by TLC analysis. After the reaction mixture was cooled to room temperature, 100 mL saturated brine were added to the mixture. The product was extracted with dichloromethane (3 × 100 ml) and the organic layers were combined. The organic solution was dried over anhydrous sodium sulfate and removed under reduced

pressure. The residue was purified by column chromatography on silica gel with petroleum ether/ dichloromethane (80:20, v/v) as eluent to afford compound **8** as a fuchsia solid (524.4 mg, 56 %). ^{1}H NMR (600 MHz, Chloroform-*d*) δ 8.12 (d, *J* = 16.6 Hz, 1H), 7.72 – 7.54 (m, 6H), 7.32 (d, *J* = 7.7 Hz, 1H), 7.14 (d, *J* = 7.9 Hz, 2H), 6.96 (d, *J* = 8.7 Hz, 4H), 4.20 (t, *J* = 5.0 Hz, 4H), 3.63 (t, *J* = 5.0 Hz, 4H), 2.46 (s, 3H), 1.46 (s, 6H).

Synthesis of **D**: Compound **8** (300.0 mg, 0.32 mmol), compound **5** (185.8 mg, 0.64 mmol), $CuSO_4$•$5H_2O$ (12.0 mg, 0.04 mmol) and sodium ascorbate (16.0 mg, 0.08 mmol) were dissolved in the mixture solution (THF/EtOH/H_2O, 60 mL, 40/10/10). The reaction mixture was stirred at room temperature for 24 hours under N_2 atmosphere. Then, the product was extracted into dichloromethane. The organic layer was dried over anhydrous Na_2SO_4 and evaporated under reduced pressure. The residue was purified by column chromatography on silica gel with CH_2Cl_2/MeOH (50:1, v/v) as eluent, and **D** was obtained as a purple dark solid (330.1 mg, 68 %). ^{1}H NMR (600 MHz, Chloroform-*d*) δ 13.08 (s, 2H), 12.19 (s, 2H), 10.90 (t, *J* = 5.7 Hz, 2H), 8.14 (d, *J* = 16.6 Hz, 2H), 7.85 (s, 2H), 7.63 – 7.50 (m, 6H), 7.33 (d, *J* = 7.7 Hz, 2H), 7.14 (d, *J* = 7.6 Hz, 2H), 6.92 (d, *J* = 8.3 Hz, 4H), 5.76 (s, 2H), 4.86 – 4.51 (m, 8H), 4.36 (t, *J* = 4.8 Hz, 4H), 2.47 (s, 3H), 2.33 – 2.15 (m, 2H), 1.77 – 1.38 (m, 14H), 1.34 – 1.15 (m, 8H), 0.91 (t, *J* = 7.3 Hz, 6H), 0.84 (t, *J* = 7.1 Hz, 6H). ^{13}C NMR (150 MHz, Chloroform-*d*) δ 173.11, 159.10, 157.05, 155.88, 154.82, 150.36, 146.32, 146.14, 139.72, 139.43, 138.92, 133.21, 132.24, 130.42, 130.23, 129.32, 128.22, 122.89, 117.03, 114.93, 106.46, 82.92, 66.77, 49.88, 45.44, 36.08, 32.96, 29.36, 26.63, 22.50, 21.57, 17.78, 13.96, 11.80. HRMS: m/z; $[M+H]^+$, calcd for $C_{68}H_{78}BF_2I_2N_{16}O_6^+$: 1517.4441; found: 1517.4439.

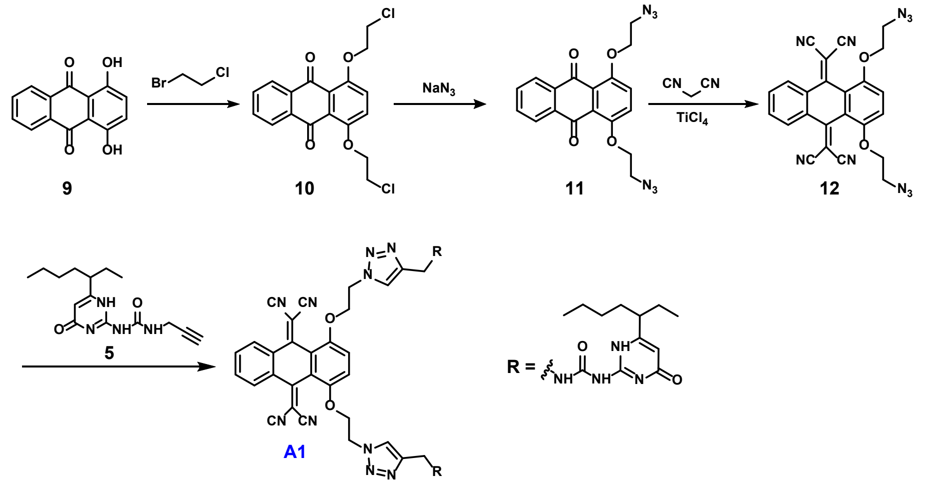

Supplementary Scheme 3 | Synthesis of compound A1.

Synthesis of compound **10**: To a 500 mL round-bottom flask were added compound **9** (720.0 mg, 3.0 mmol), 1-bromo-2-chloroethane (946.5 mg, 6.6 mmol), Cs_2CO_3 (2.93 g, 9.0 mmol) and acetonitrile (200 mL), and then the reaction mixture was refluxed for 24 hours. After the reaction mixture was cooled to room temperature, 200 mL saturated brine were added to the mixture. The product was extracted with ethyl acetate (3 × 200 ml) and the organic layers were combined. The organic solution was dried over anhydrous sodium sulfate and removed under reduced pressure. The residue was purified by column

chromatography on silica gel with petroleum ether/ dichloromethane (30:70, v/v) as eluent to afford compound **10** as an orange solid (679.3 mg, 62 %). ^{1}H NMR (600 MHz, Chloroform-*d*) δ 8.08 (dd, *J* = 5.7, 3.3 Hz, 2H), 7.66 (dd, *J* = 5.8, 3.2 Hz, 2H), 7.29 (s, 2H), 4.30 (t, *J* = 6.0 Hz, 4H), 3.90 (t, *J* = 5.9 Hz, 4H).

Synthesis of compound **11**: NaN_3 (247.0 mg, 3.8 mmol) was added to a solution of 10 mL of DMF of compound **10** (547.8 mg, 1.5 mmol). The reaction mixture was heated to 100 °C for 12 hours and then cooled to room temperature. 50 mL of ice water was added to the reaction mixture, and a large amount of yellow solid was precipitated. Compound **11** (556.2 mg, 98%) was obtained by filtration. ^{1}H NMR (600 MHz, Chloroform-*d*) δ 8.15 (dd, *J* = 5.8, 3.3 Hz, 2H), 7.71 (dd, *J* = 5.8, 3.3 Hz, 2H), 7.34 (s, 2H), 4.26 (t, *J* = 5.0 Hz, 4H), 3.76 (t, *J* = 5.0 Hz, 4H).

Synthesis of compound **12**: Malononitrile (198.2 mg, 3.0 mmol) was added to 50 mL dichloromethane solution of compound **11** (378.3 mg, 1.0 mmol). $TiCl_4$ (0.6 mL, 5.5 mmol) was slowly added dropwise under N_2 atmosphere, and the solution changed from pale yellow to dark yellow. Then, pyridine (0.16 mL, 1.9 mmol) was added dropwise under an ice bath with the formation of a yellow solid. The mixture was heated to reflux for 24 hours. The mixture was poured into ice water and extracted with dichloromethane (3 × 100 mL). The combined organic layers were dried with anhydrous magnesium sulfate and concentrated in vacuo. The residue was purified by column chromatography on silica gel with dichloromethane as eluent to afford compound **12** as an orange solid (223.0 mg, 47 %). ^{1}H NMR (600 MHz, Chloroform-*d*) δ 8.07 – 8.19 (m, 2H), 7.80 – 7.57 (m, 2H), 7.42 – 7.08 (m, 2H), 4.70 – 4.15 (m, 4H), 4.08 – 3.41 (m, 4H).

Synthesis of compound **A1**: Compound **12** (237.2 mg, 0.5 mmol), compound **5** (290.3 mg, 1.0 mmol), $CuSO_4{\cdot}5H_2O$ (15.0 mg, 0.05 mmol) and sodium ascorbate (20.0 mg, 0.1 mmol) were dissolved in the mixture solution (THF/EtOH/H_2O, 60 mL, 40/10/10). The reaction mixture was stirred at room temperature for 24 hours under N_2 atmosphere. Then, the product was extracted into dichloromethane. The organic layer was dried over anhydrous Na_2SO_4 and evaporated under reduced pressure. The residue was purified by column chromatography on silica gel with CH_2Cl_2/MeOH (50:1, v/v) as eluent, and **A1** was obtained as an orange solid (274.3 mg, 52 %). ^{1}H NMR (600 MHz, Chloroform-*d*) δ 13.09 (s, 2H), 12.10 (s, 2H), 10.83 (s, 2H), 8.26 (s, 2H), 8.11 – 7.99 (m, 2H), 7.62 (s, 2H), 7.17 – 6.96 (m, 2H), 5.90 – 5.65 (m, 2H), 4.88 – 4.23 (m, 14H), 2.25 (s, 2H), 1.37 – 1.66 (m, 6H), 1.04 – 1.32 (m, 8H), 0.69-0.89 (m, 12H). ^{13}C NMR (150 MHz, Chloroform-*d*) δ 181.78, 173.07, 158.98, 157.06, 155.82, 154.79, 152.65, 149.04, 145.84, 145.50, 132.99, 132.58, 127.38, 126.23, 124.83, 124.35, 123.41, 121.44, 120.01, 119.06, 113.72, 113.46, 106.44, 86.14, 69.13, 66.79, 49.62, 49.28, 46.12, 35.65, 33.28, 31.66, 29.32, 27.12, 22.72, 22.53, 13.45, 11.74. HRMS: m/z; $[M+H]^+$, calcd for $C_{54}H_{59}N_{18}O_6^+$: 1055.4865; found: 1055.4869.

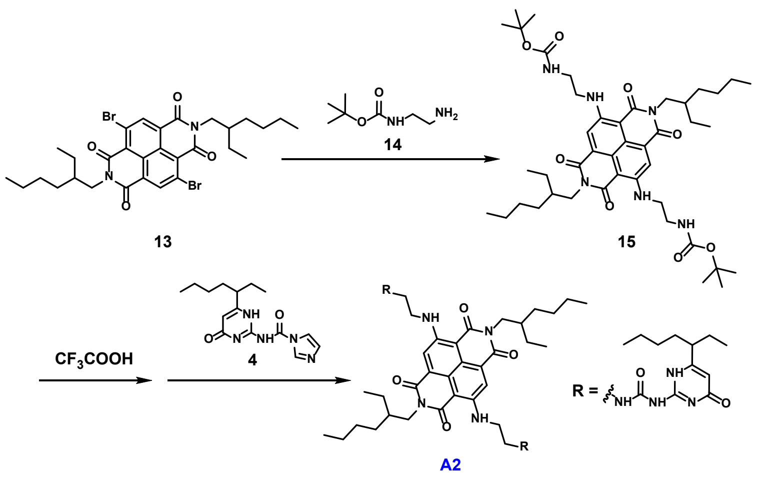

Supplementary Scheme 4 | Synthesis of compound A2.

Synthesis of compound **15**: Compound **13** (100 mg, 0.15 mmol) and compound **14** (0.85 g, 4.5 mmol) were mixed in a 100 mL thick wall round-bottom flask. The temperature was raised to 120 °C, and the mixture gradually melted as the temperature increased. The reaction was carried out for 1 hour at 120 °C. After the reaction mixture was cooled to room temperature, 100 mL dichloromethane was added to dissolve and washed three times with saturated brine (3 × 100 mL). The organic phase was collected and dried over anhydrous sodium sulfate. After filtration, the dichloromethane was removed under reduced pressure. The obtained crude product was separated through a column chromatography CH_2Cl_2/MeOH (20:1, v/v) and purified to obtain product compound **15** as a purple solid (93.2 mg, 77%). ^{1}H NMR (600 MHz, Chloroform-*d*) δ 9.46 (s, 2H), 8.11 (s, 2H), 4.99 (s, 2H), 4.13 – 4.05 (m, 4H), 3.64 (s, 4H), 3.53 (s, 4H), 2.01 (s, 2H), 1.87 (s, 4H), 1.44 – 1.54 (m, 4H), 1.46 (s, 18H), 1.26 – 1.32 (m, 16H), 0.88 – 0.92 (m, 12H).

Synthesis of compound **A2**: Compound **15** (100.0 mg, 0.11 mmol) was dissolved in 95% TFA of dichloromethane solution, then stirred at room temperature for 4 hours and solvent was removed under reduced pressure. The product was used for the next step without puritification. Then to a solution of compound **15** in 5 mL anhydrous trichloromethane was added compound **4** (105.0 mg, 0.15 mmol), then the mixture was stirred at room temperature under N_2 atmosphere until compound **15** had been completely consumed by TLC analysis. After the reaction was completed, the resulting mixture was washed with 2 M HCl, saturated aqueous $NaHCO_3$, brine and dried over anhydrous Na_2SO_4. Evaporation of the solvent under reduced pressure and the further purification was carried out by column chromatography using $CHCl_3$/CH_3OH (100:5, v/v) as eluent to give a purple solid (79.4 mg, 67 %). ^{1}H NMR (600 MHz, Chloroform-*d*) δ 13.13 (s, 2H), 11.98 (s, 2H), 10.69 (s, 2H), 9.57 (s, 2H), 8.30 (s, 2H), 5.77 (s, 2H), 4.07 (dt, *J* = 35.1, 8.5 Hz, 4H), 3.80 (q, *J* = 6.6 Hz, 4H), 3.66 – 3.59 (m, 4H), 2.31 (q, *J* = 7.5 Hz, 2H), 1.95 – 1.87 (m, 2H), 1.53 – 1.70 (m, 12H), 1.24 – 1.34 (m, 20H), 0.84 – 0.92 (m, 24H). ^{13}C NMR (100 MHz, Chloroform-*d*) δ 173.12, 166.59, 164.17, 157.90, 155.76, 154.77, 148.36, 127.02, 122.30, 118.71, 106.34, 103.06, 45.36, 44.11, 42.32, 39.75, 37.91, 32.85, 30.88, 29.42, 28.81, 26.59, 24.12, 23.23, 22.64, 14.23, 14.03, 11.84, 10.76. HRMS: m/z; $[M+H]^+$, calcd for $C_{58}H_{85}N_{12}O_8^-$: 1077.6613; found: 1077.6598.

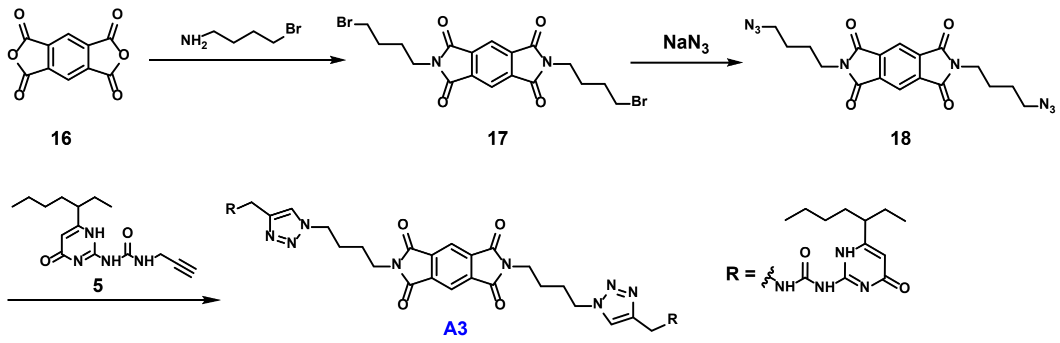

Supplementary Scheme 5 | Synthesis of compound A3.

Synthesis of compound **17**: Compound **16** (500.0 mg, 2.3 mmol), 4-bromobutan-1-amine (1.05 g, 6.9 mmol) and triethylamine (1 mL) were dissolved in 10 mL glacial acetic acid, and then the reaction mixture was refluxed for 12 hours. After the reaction mixture was cooled to room temperature, a large amount of solid was precipitated, which was filtered and washed with water and methanol to obtain an off-white compound **17** (1.05 g, 94%). ^{1}H NMR (600 MHz, Chloroform-*d*) δ 8.28 (s, 2H), 3.79 (t, *J* = 6.6 Hz, 4H), 3.45 (t, *J* = 6.1 Hz, 4H), 2.03 – 1.79 (m, 8H).

Synthesis of compound **18**: NaN_3 (247.0 mg, 3.8 mmol) was added to a solution of 10 mL of DMF of compound **17** (729.2 mg, 1.5 mmol). The reaction mixture was heated to 100 °C for 12 hours and then cooled to room temperature. 50 mL of ice water was added to the reaction mixture, and a large amount of solid was precipitated. Compound **18** (591.0 mg, 96%) was obtained by filtration as a white solid. ^{1}H NMR (600 MHz, Chloroform-*d*) δ 8.27 (s, 2H), 3.78 (t, *J* = 7.1 Hz, 4H), 3.34 (t, *J* = 6.7 Hz, 4H), 1.90 – 1.75 (m, 4H), 1.73 – 1.54 (m, 4H).

Synthesis of compound **A3**: Compound **18** (205.2 mg, 0.5 mmol), compound **5** (290.3 mg, 1.0 mmol), $CuSO_4$•$5H_2O$ (15.0 mg, 0.05 mmol) and sodium ascorbate (20.0 mg, 0.1 mmol) were dissolved in the mixture solution (THF/EtOH/H_2O, 60 mL, 40/10/10). The reaction mixture was stirred at room temperature for 24 hours under N_2 atmosphere. Then, the product was extracted into dichloromethane. The organic layer was dried over anhydrous Na_2SO_4 and evaporated under reduced pressure. The residue was purified by column chromatography on silica gel with CH_2Cl_2/MeOH (20:1, v/v) as eluent, and **A3** was obtained as a white solid (232.9 mg, 47 %). ^{1}H NMR (600 MHz, Chloroform-*d*) δ 13.09 (s, 2H), 11.97 (d, *J* = 61.9 Hz, 2H), 10.73 (s, 2H), 7.73 (s, 2H), 5.78 (s, 2H), 4.09 – 4.64 (m, 8H), 3.49 – 3.84 (m, 6H), 2.30 (s, 2H), 1.95 (s, 2H), 1.47 – 1.78 (m, 12H), 1.21 – 1.30 (m, 8H), 0.88 – 0.81 (m, 12H). ^{13}C NMR (100 MHz, CHLOROFORM-*D*) δ 172.99, 166.24, 166.02, 156.86, 155.85, 153.91, 136.07, 118.37, 118.13, 106.33, 49.37, 45.22, 37.44, 35.71, 32.88, 29.33, 26.98, 26.64, 25.07, 22.53, 13.95, 11.68. HRMS: m/z; $[M+H]^+$, calcd for $C_{48}H_{62}N_{16}O_8^+$: 991.5015; found: 991.5005.

3. The preparation and properties of supramolecular PS.

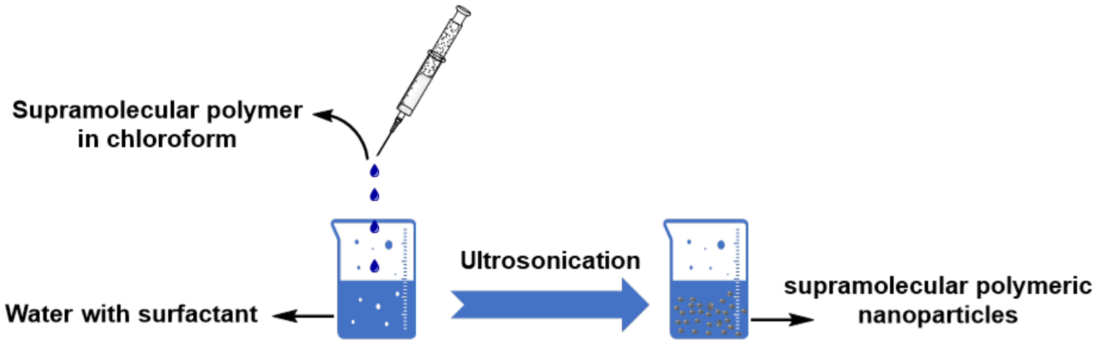

Supplementary Fig. 1 | The scheme of the preparation of supramolecular photodynamic agents.

The preparation of the quadruple hydrogen bonded supramolecular polymeric photosensitizers (Fig. 1): A mixture of **D** and the electron acceptor (2 mg, molar ratio 4:6) in 200 μL chloroform was quickly added into deionized water with 1 mg/mL Pluronic F127 as surfactant (5 mL). The resulting mixture was sonicated for 35 min by ultrasonic cell disruptor. After centrifuge-washing with deionized water three times, the water-dispersible nanoparticles were obtained.

a)

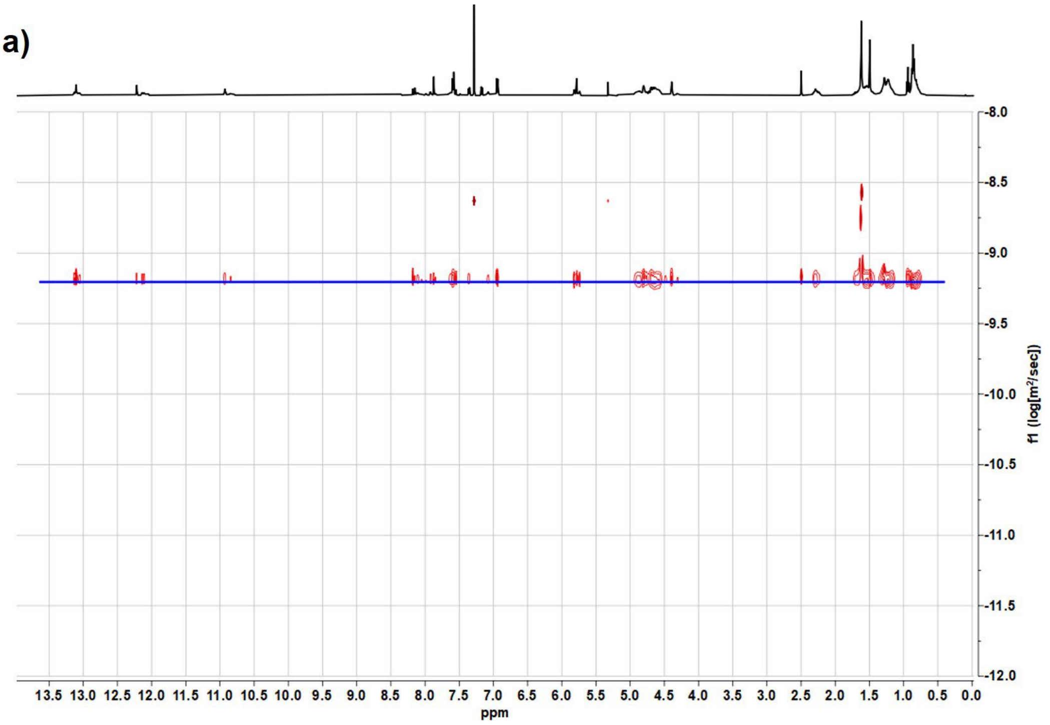

b)

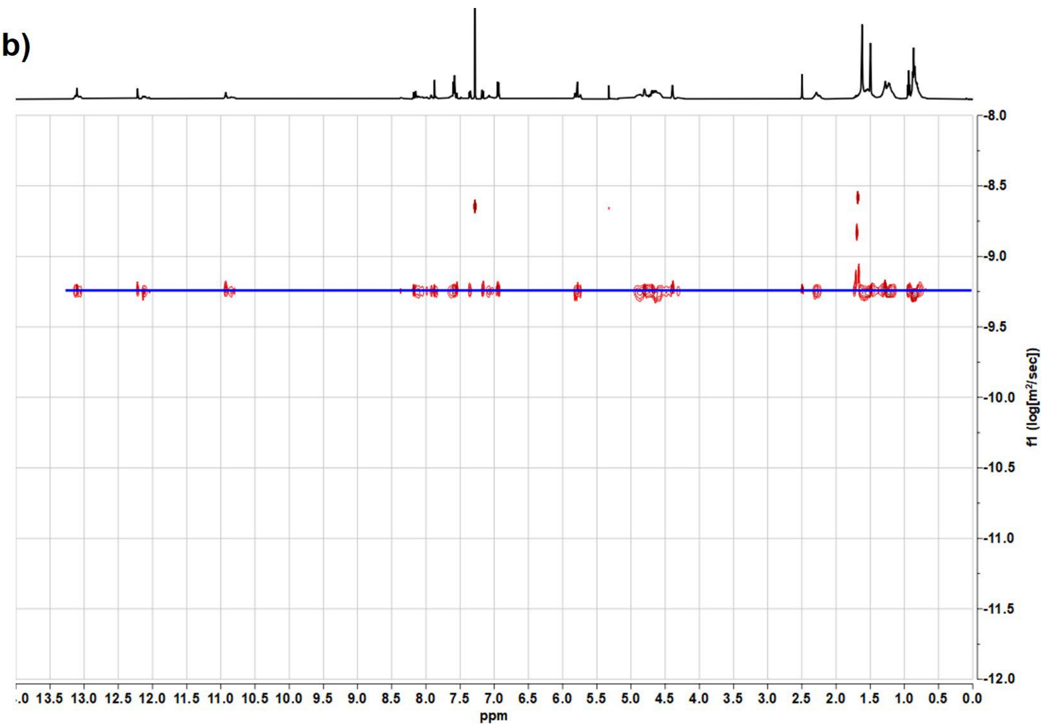

c)

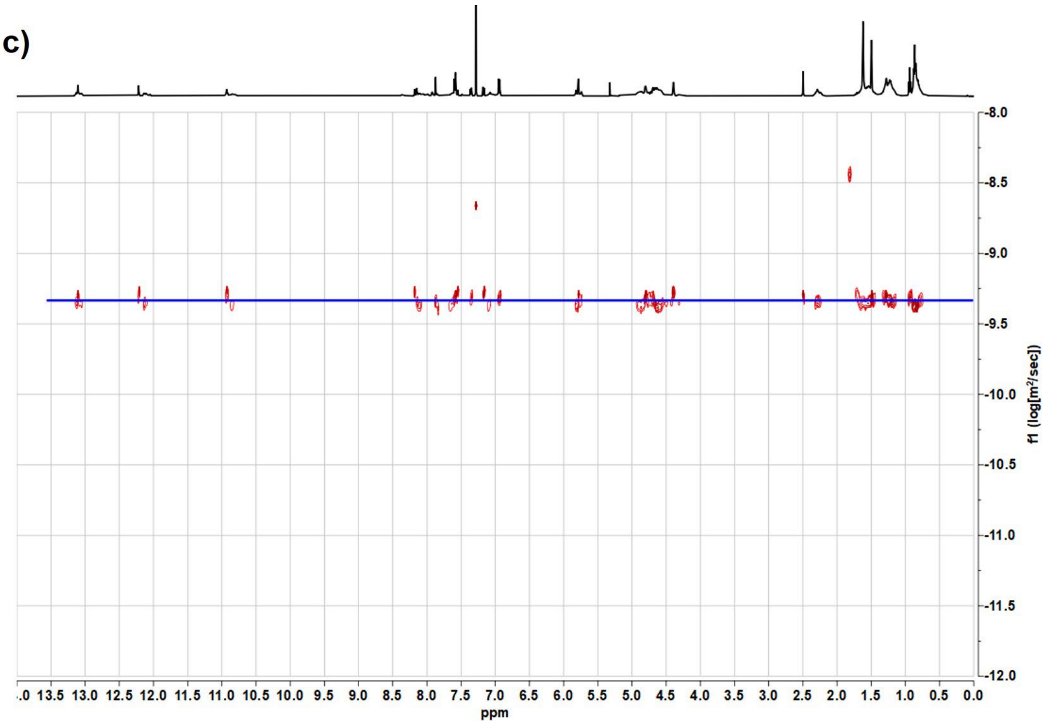

d)

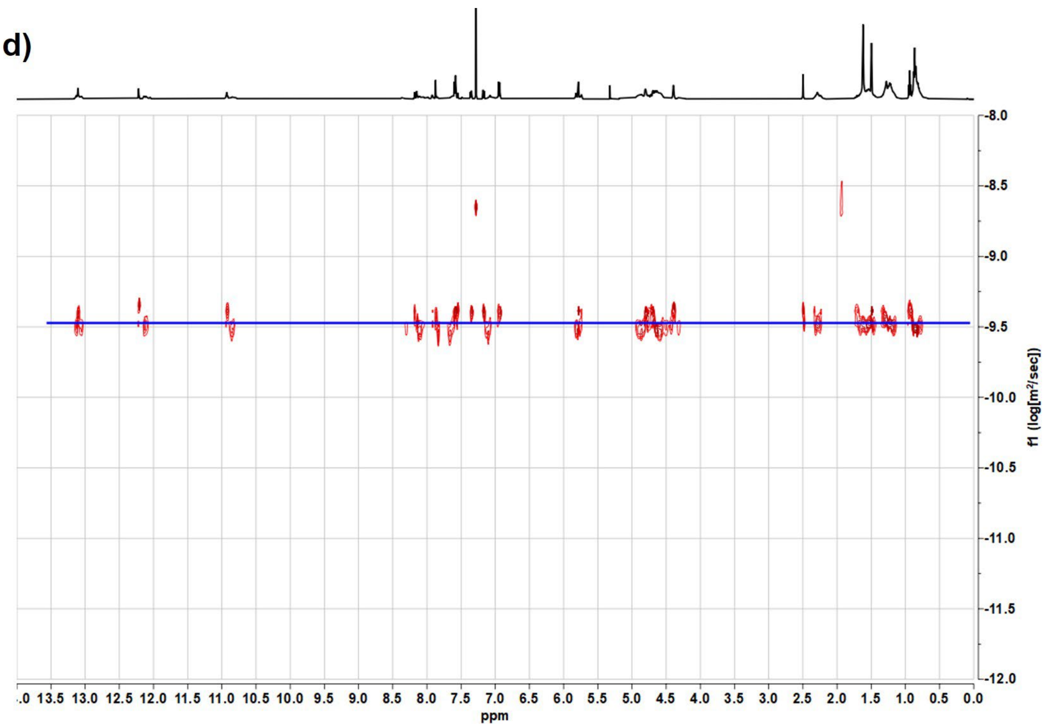

e)

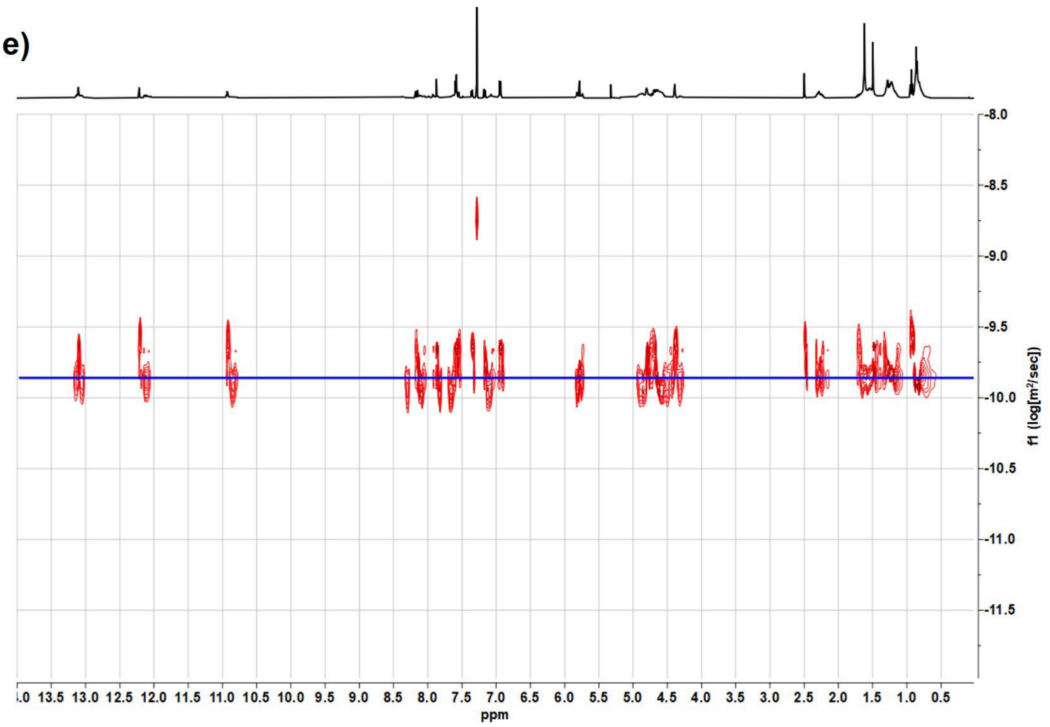

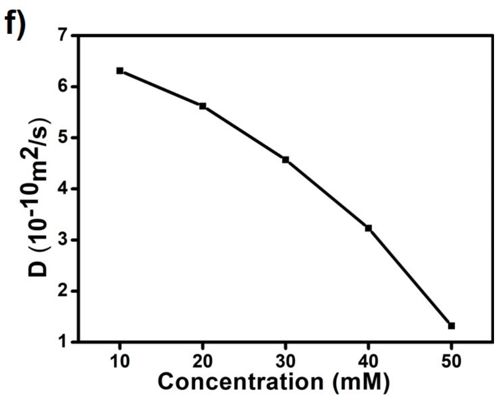

Supplementary Fig. 2 | DOSY NMR. DOSY NMR (700 MHz, 298 K) spectra of mixture of **D** and **A1** (molar ratio 4:6) at a) 10 mM; b) 20 mM; c) 30 mM; d) 40 mM; e) 50 mM. f) DOSY (500 MHz, 298 K) plots of solutions in $CDCl_3$ of mixture of **D** and **A1** at various concentrations.

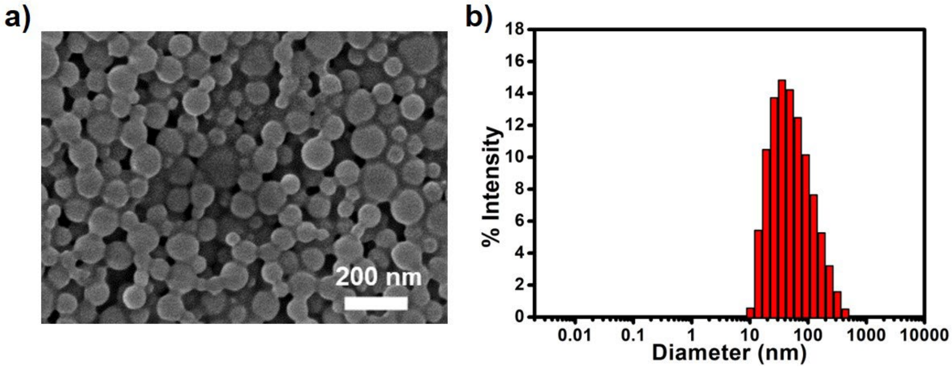

Supplementary Fig. 3 | Morphology properties of DA1. a) SEM image of **DA1**. The experiment was repeated three times independently, with similar results. Scale bar: 200 nm. b) The particle size distribution plots of **DA1** from DLS. The experiment was repeated three times independently, with similar results.

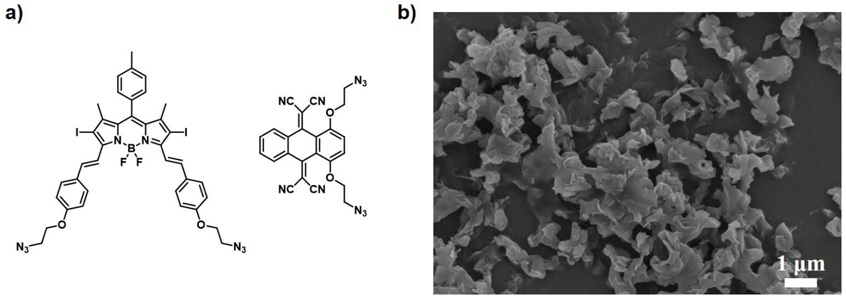

Supplementary Fig. 4 | Morphology properties of assembly without UPy. a) Structures of **D** and **A1** without UPy. b) The SEM image of the assembly without quadruple hydrogen-bonds. Preparation method: A mixture of **D** and **A1** without UPy (2 mg, molar ratio 4:6) in 200 μL chloroform was quickly added into deionized water with 1 mg/mL Pluronic F127 as surfactant (5 mL). The resulting mixture was sonicated for 35 min by ultrasonic cell disruptor. After centrifuge-washing with deionized water

three times, the water-dispersible nanoparticles were obtained. The experiment was repeated three times independently, with similar results. Scale bar: 1 μm.

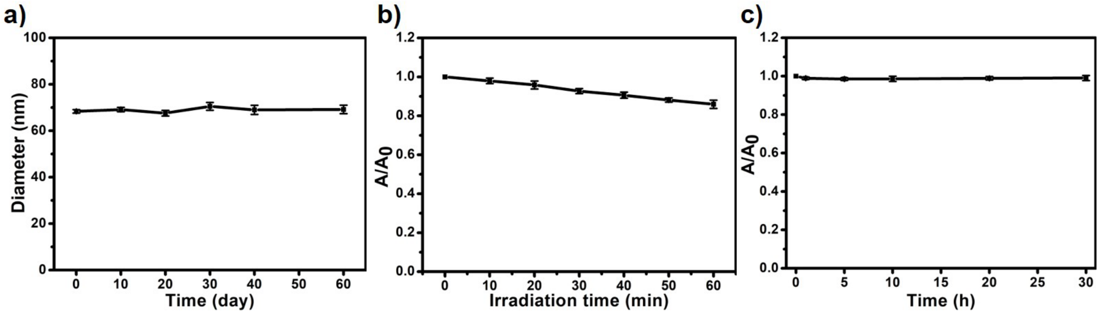

Supplementary Fig. 5 | Stability test of DA1. a) Hydrodynamic diameter of **DA1** in PBS buffer in different days. b) The A/A_0 at 665 nm of **DA1** at different times under the illumination of LED light (660 nm, 40 mW/cm^2). c) The A/A_0 at 665 nm of **HG** in complete medium at different times. Data in a-c) are presented as mean ± s.d. derived from n = 4 independent samples.

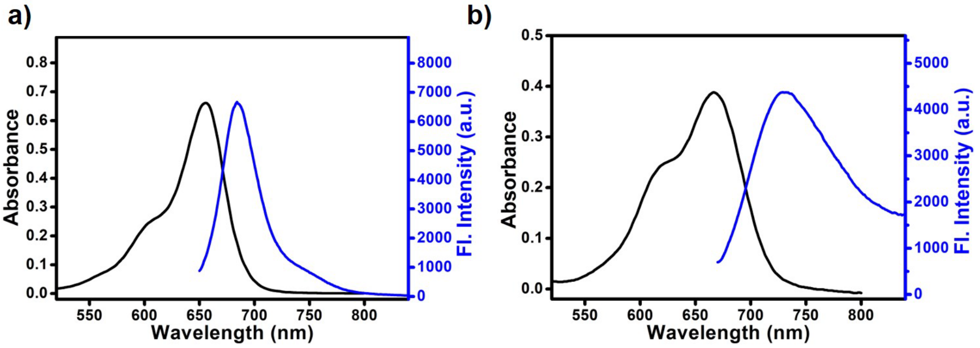

Supplementary Fig. 6 | Photophysical properties of D and DA1. The absorption and fluorescence spectra of a) **D** (10 μM) in DCM and b) **DA1** (10 μM) dispersed in water.

4. The evaluation of ROS generation ability.

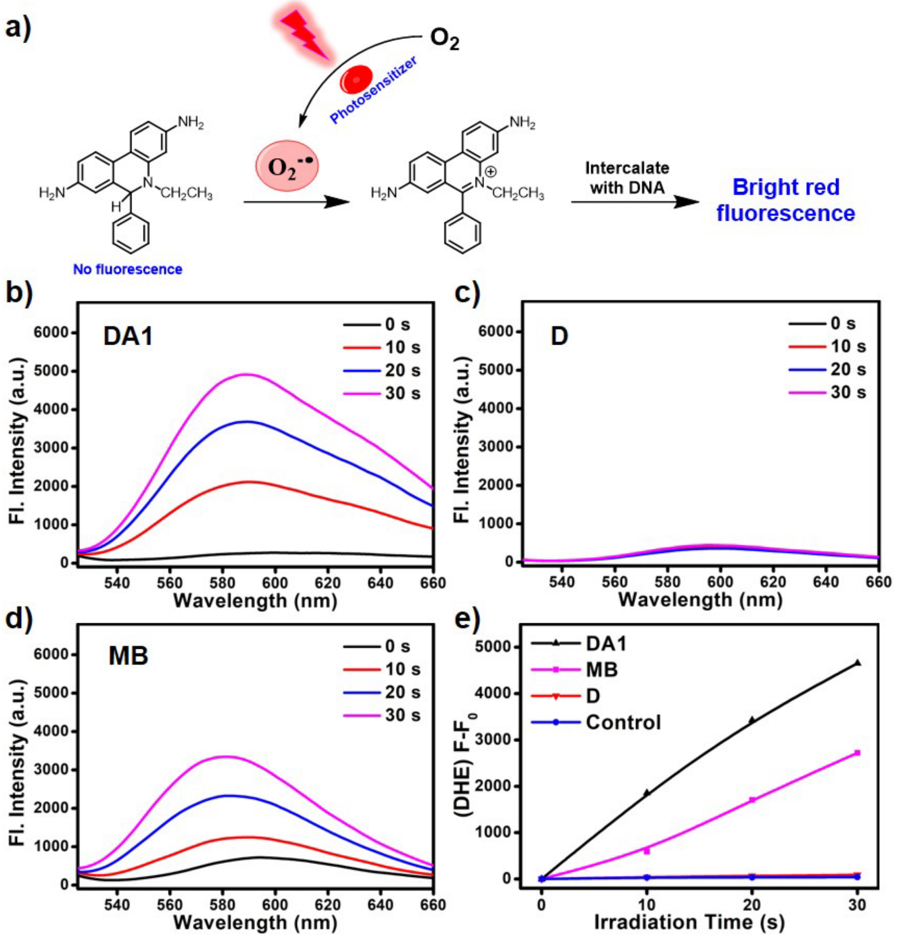

Supplementary Fig. 7 | The $O_2^{-\bullet}$ generation of PSs. a) The mechanism of droethidium (DHE) as the scavenger monitors $O_2^{-\bullet}$ in the solution. The fluorescence spectra of DHE (40 μM, excitation at 510 nm, detection from 525 nm to 660 nm) containing 500 μg/mL ctDNA after irradiation (660 nm, 20 mW/cm^2) for different time in the presence of b) **DA1** (10 μM) dispersed in PBS; c) **D** (10 μM) in PBS containing 20% DMF and d) MB in PBS. e) Plots of ΔFl. (F-F$_0$) of DHE at 580 nm upon light irradiation (660 nm, 20 mW/cm^2) for different time intervals in the presence of **DA1**, MB or **D**.

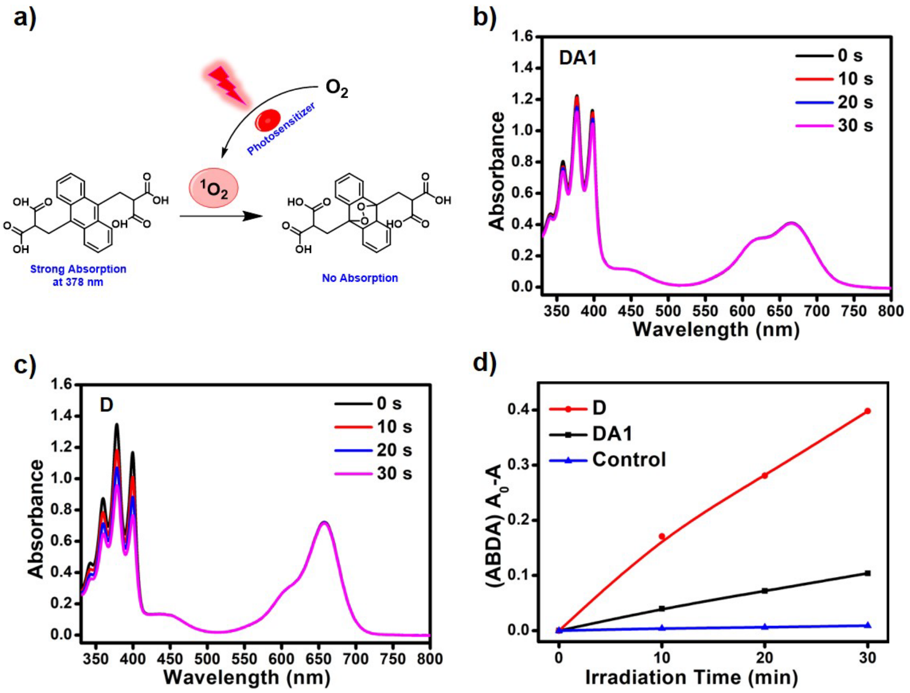

Supplementary Fig. 8 | The $^1O_2$ generation of PSs. a) The mechanism of 9,10-anthracenediyl-bis(methylene)-dimalonic acid (ABDA) as the $^1O_2$ scavenger monitors singlet oxygen generation in the solution. The absorption spectra of ABDA (30 μM) after irradiation (660 nm, 20 mW/cm^2) for different time in the presence of b) **DA1** (10 μM) dispersed in PBS and c) **D** (10 μM) in PBS containing 20% DMF. d) Plots of ΔAbs (A_0-A) of ABDA at 378 nm upon light irradiation (660 nm, 20 mW/cm^2) for different time intervals in the presence of **D** or **DA1**.

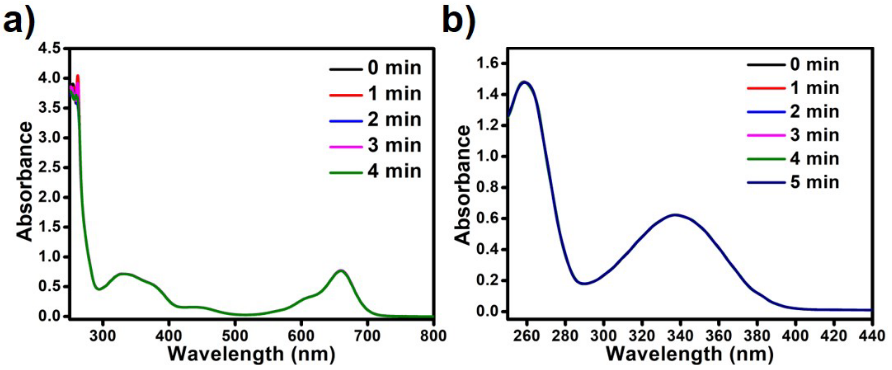

Supplementary Fig. 9 | The oxidation of NADH of monomer and control group. a) The absorption spectra of NADH (100 μM) after irradiation (660 nm, 20 mW/cm^2) for different time in the presence of **D** (10 μM) in PBS containing 20% DMF. b) The absorption spectra of NADH (100 μM) after irradiation (660 nm, 20 mW/cm^2) for different time in PBS.

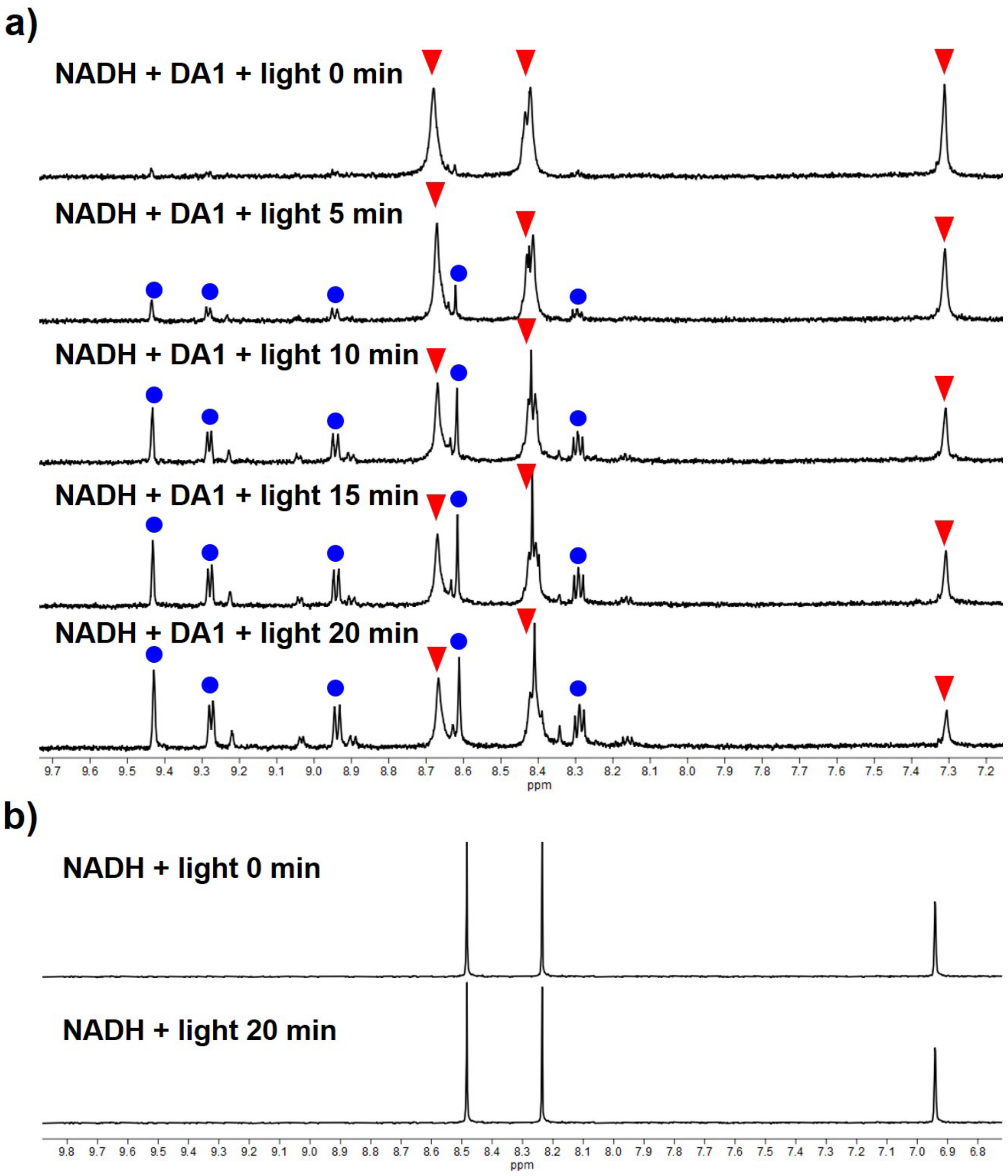

Supplementary Fig. 10 | The characterization of the oxidation of NADH by ^{1}H NMR. a) ^{1}H NMR of NADH (2.0 mM) in D_2O containing **DA1** (0.1 mM) after irradiating for different time (660 nm, 20 mW/cm^2). Peaks labeled with red triangles represent NADH and peaks labeled with blue circles represent NAD^+. b) ^{1}H NMR of NADH (2.0 mM) in D_2O after irradiating for different time (660 nm, 20 mW/cm^2).

5. The study of photoinduced electron transfer between D and A1.

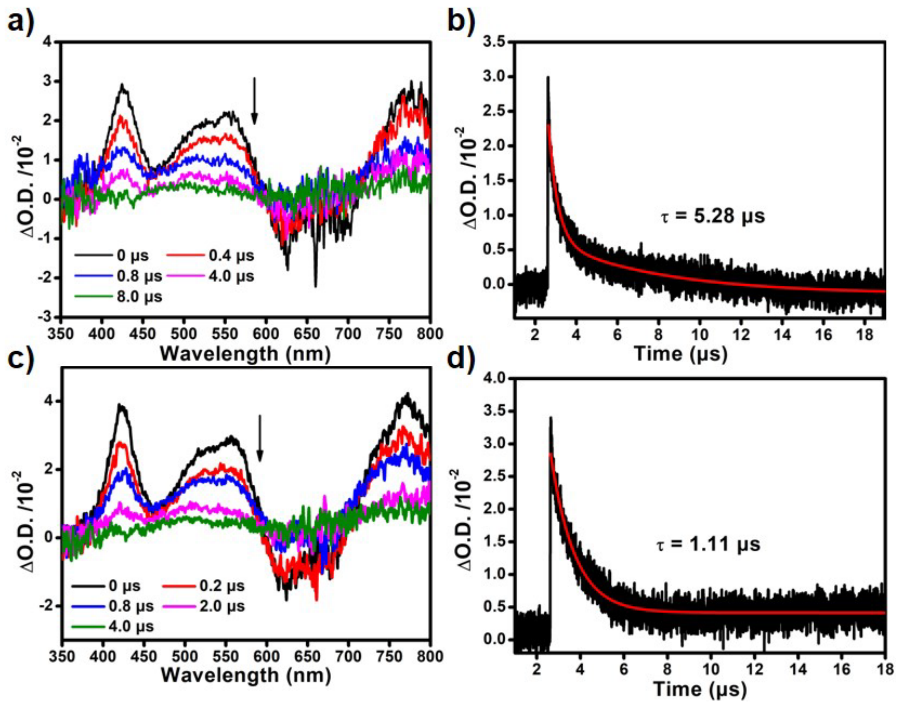

Supplementary Fig. 11 | The characterization of the electron transfer by time-resolved transient difference absorption. Time-resolved transient difference absorption of **D** in the a) supramolecular polymer without electron acceptor and c) **DA1** dispersed in water (20 mM). Decay trace of **D** in the b) supramolecular polymer without electron acceptor and d) **DA1** dispersed in water (20 mM). Excitation wavelength: 660 nm; Monitoring wavelength: 550 nm.

$$\Delta G = e\left[E_{D^{+\bullet}/D} - E_{A/A^{-\bullet}} - E_{00}\right] - \frac{e^2}{4\pi\varepsilon_s\varepsilon_0 R_{cc}} - \frac{e^2}{8\pi\varepsilon_0}\left(\frac{1}{r^+}+\frac{1}{r^-}\right)\left(\frac{1}{\varepsilon_{ref}}-\frac{1}{\varepsilon_s}\right)$$
$$\approx e\left[E_{D^{+\bullet}/D} - E_{A/A^{-\bullet}} - E_{00}\right] \quad (1)$$

Gibbs-free energy changes of the electron transfer from D to **A1** can be calculated using Weller eqs (1), where $E_{D+\bullet/D}$ is onset potential for one electron oxidation in the electron donor unit; $E_{A/A-\bullet}$ is the onset potential for one electron reduction of the electron acceptor unit; E_{00} can be estimated at the intersection between the normalized absorbance and emission spectra of **D** after converting the wavelength axis to an energy scale.[3-4]

Electrochemical measurements show that **D** has oxidation potentials of +0.440 V vs Fc/Fc$^+$ (Fig. 12a), and the **A1** has reduction potentials of -1.183 V vs Fc/Fc$^+$ (Fig. 12b). The excited state energy E_{00} is estimated to be 1.85 eV (Fig. 12c). Rehm−Weller theory calculations show that the ΔG is -22.2 KJ/mol, which indicates photoinduced electron transfer between excited-state **D** to **A1** is thermodynamically feasible.

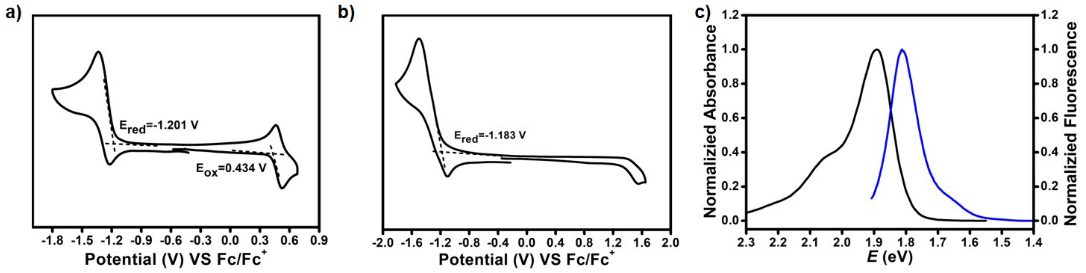

Supplementary Fig. 12 | Calculation of Gibbs-free energy. Cyclic voltammogram of a) **D** and b) **A1** in DCM with 0.1 M $(n\text{-Bu})_4N^+PF_6^-$ as a supporting electrolyte, Ag/Ag^+ as a reference electrode, platinum-carbon electrode as a working electrode and Pt wire as a counter electrode; scan rate, 100 mVs^{-1}; Fc/Fc^+ was used as an external reference. c) Normalized absorbance and emission spectra of **D** in DCM after converting the wavelength axis to an energy scale.

6. The expansion of electron acceptors.

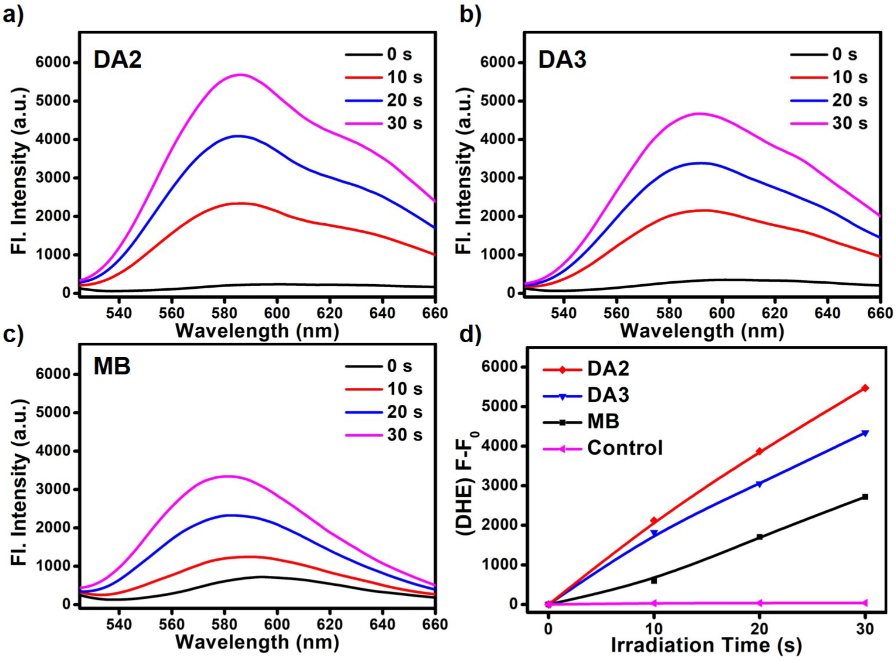

Supplementary Fig. 13 | The $O_2^{-\bullet}$ generation of DA2 and DA3. The fluorescence spectra of DHE (40 μM, excitation at 510 nm, detection from 525 nm to 660 nm) containing 500 μg/mL ctDNA after irradiation (660 nm, 20 mW/cm^2) for different time in the presence of 10 μM a) **DA2**; b) **DA3** and c) MB in PBS. d) Plots of ΔFl. (F-F_0) of DHE at 580 nm upon light irradiation (660 nm, 20 mW/cm^2) for different time intervals in the presence of **DA2**, **DA3** **or** MB.

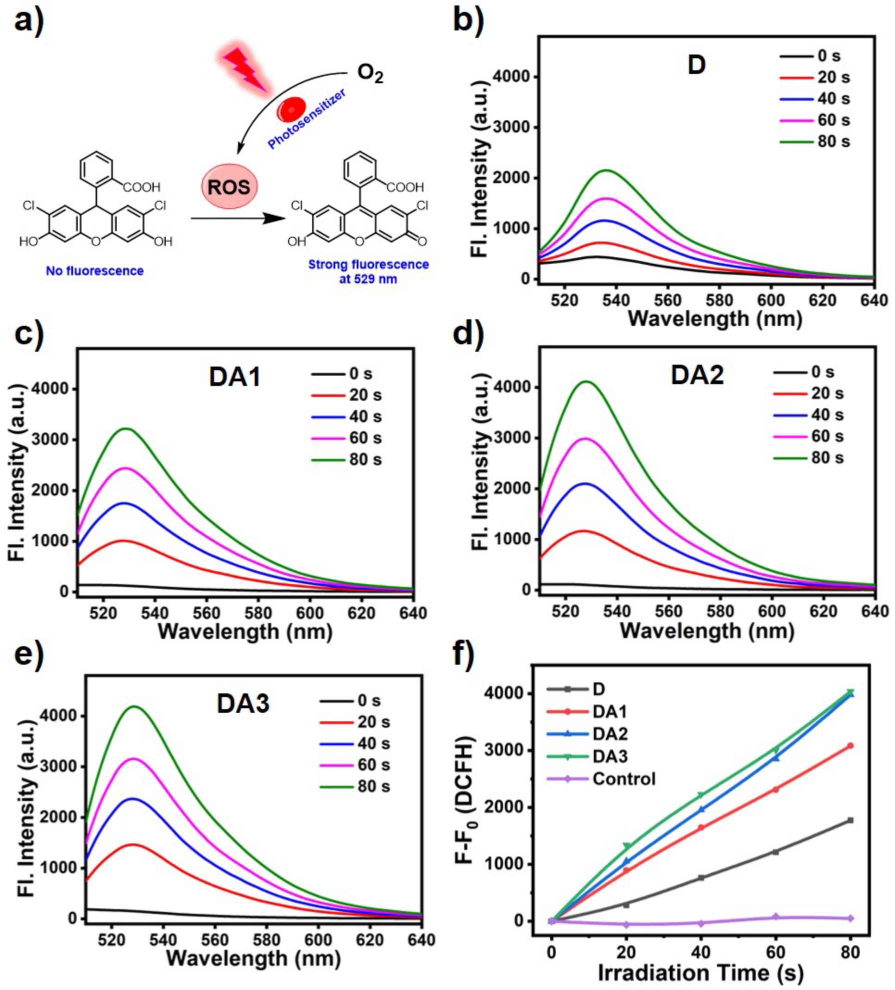

Supplementary Fig. 14 | The ROS generation of PSs. a) The mechanism of 2',7'-dichlorodihydrofluorescein (DCFH) as the scavenger monitors any general types of ROS in the solution. b) The fluorescence spectra of 2',7'-dichlorodihydrofluorescein (DCFH, 40 μM, excitation at 500 nm, detection from 510 nm to 640 nm) after irradiation (660 nm, 20 mW/cm^2) for different time in the presence of 10 μM a) **D** in DMF; c) **DA1**; d) **DA2** and e) **DA3** dispersed in PBS. f) Plots of ΔFl. ($F-F_0$) of DCFH at fluorescence emission maxima upon light irradiation (660 nm, 20 mW/cm^2) for different time intervals in the presence of **D**, **DA1**, **DA2** or **DA3**.

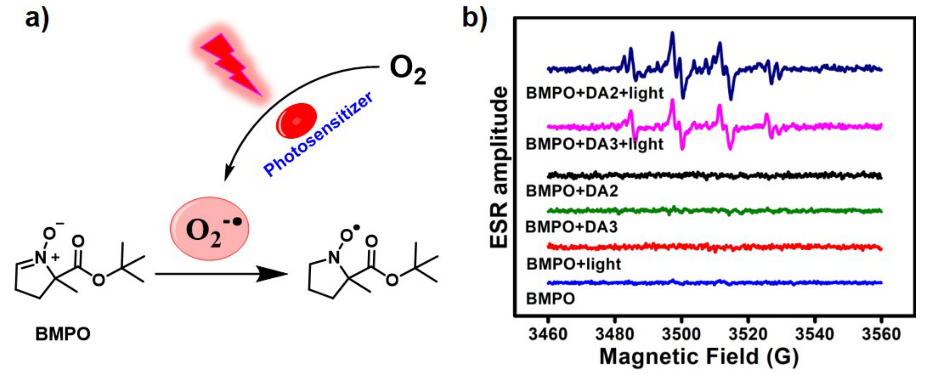

Supplementary Fig. 15 | The characterization of $O_2^{-\bullet}$ by ESR spectra. a) The mechanism of BMPO as the spin trapper to detect the $O_2^{-\bullet}$. b) ESR spectra to detect $O_2^{-\bullet}$ generated by **DA2** and **DA3** (0.5 mM) under illumination, using BMPO (25 mM) as a spin trap.

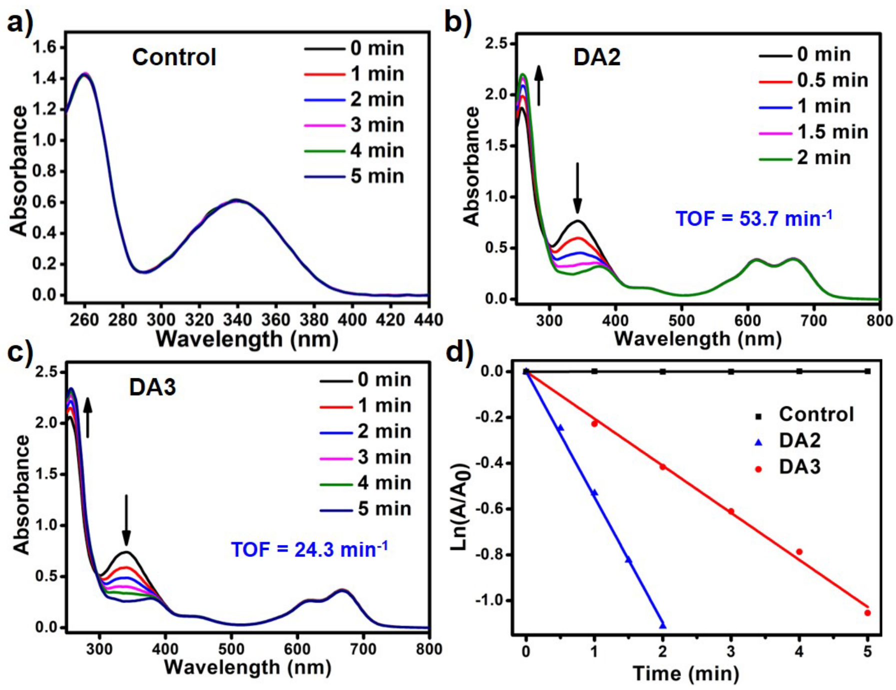

Supplementary Fig. 16 | The oxidation of NADH by DA2 and DA3. a) The absorption spectra of NADH (100 μM) after irradiation (660 nm, 20 mW/cm^2) for different time in PBS. b) The absorption spectra of NADH (100 μM) after irradiation (660 nm, 20 mW/cm^2) for different time in the presence of 10 μM b) **DA2**; c) **DA3** dispersed in PBS. d) Plots of $\ln A/A_0$ of NADH at 339 nm for different time intervals.

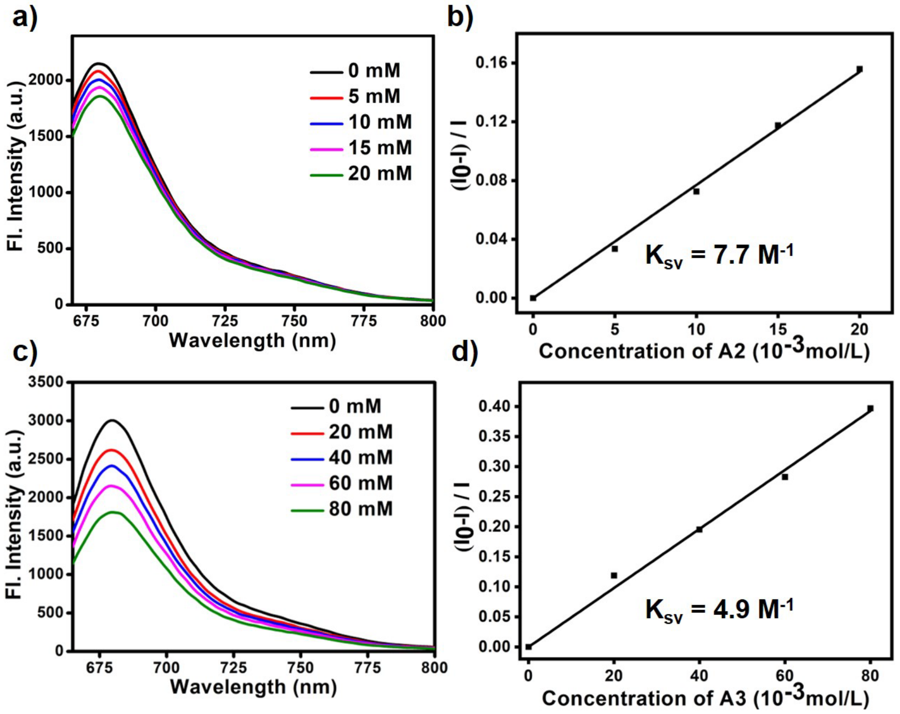

Supplementary Fig. 17 | Stern–Volmer quenching experiments. The emission spectra of **D** (1.0×10^{-5} M) at 25 ℃ in DMF with increasing amounts of a) **A2** (0 – 20 mM) and c) **A3** (0 – 80 mM) under excitation at 650 nm. The Stern–Volmer plots for the fluorescence quenching of **D** by b) **A2** and d) **A3** in acetonitrile at 25 ℃ (I_0 is the fluorescence intensity of **D** in the absence of electron acceptor, I is the fluorescence intensity in the presence of electron acceptor at the different concentrations).

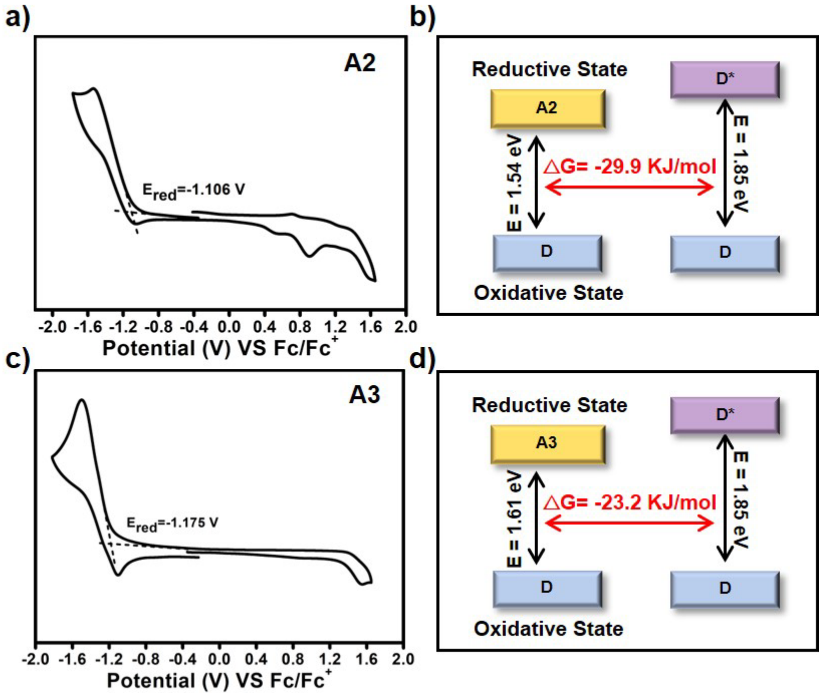

Supplementary Fig. 18 | The characterization of electron transfer by electrochemical testing. Cyclic voltammogram of a) **A2** and c) **A3** in DCM with 0.1 M $(n\text{-}Bu)_4N^+PF_6^-$ as a

supporting electrolyte, Ag/Ag^{+} as a reference electrode, platinum-carbon electrode as a working electrode and Pt wire as a counter electrode; scan rate, 100 mVs^{-1}; Fc/Fc^{+} was used as an external reference. Gibbs free energy of electron transfer from **D** to b) **A2** and d) **A3**.

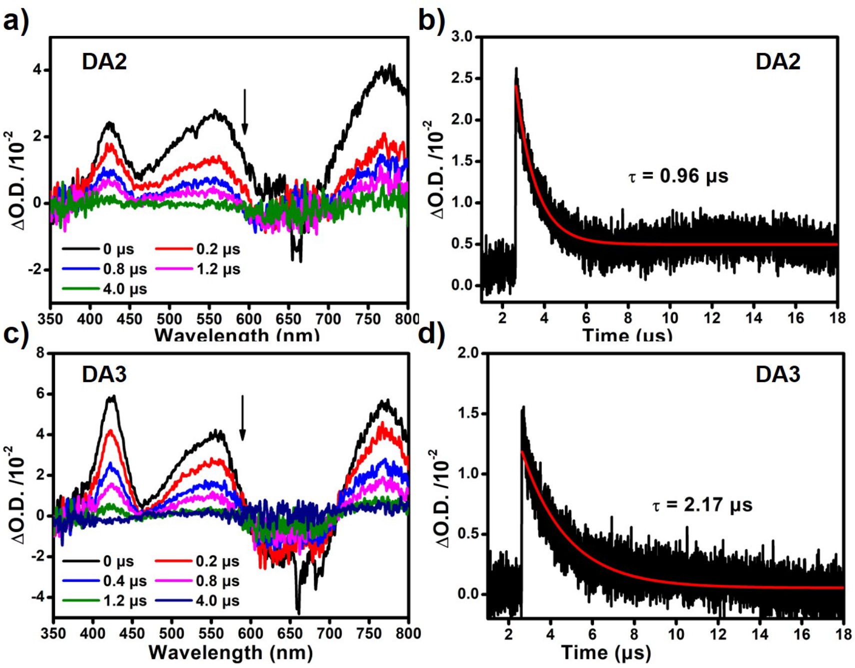

Supplementary Fig. 19 | The characterization of electron transfer by time-resolved transient difference absorption. Time-resolved transient difference absorption of **D** in the a) **DA2** and c) **DA3** dispersed in water (20 mM). Decay trace of **D** in the b) **DA2** and d) **DA3** dispersed in water (20 mM). Excitation wavelength: 660 nm; Monitoring wavelength: 550 nm.

7. Experimental data in vitro and in vivo

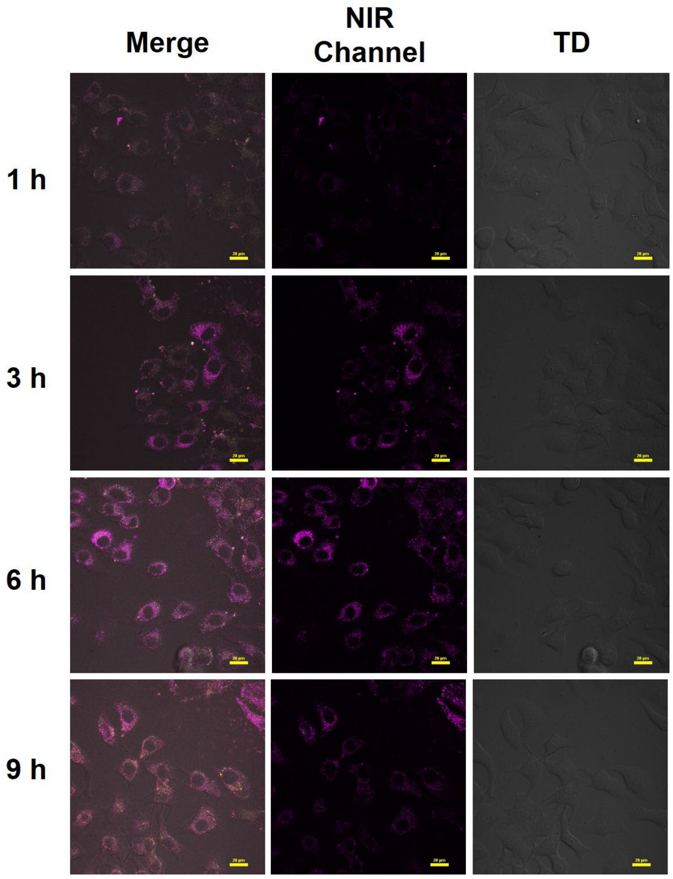

Supplementary Fig. 20 | Cellular uptake of DA1. CLSM images of HeLa cells incubated with **DA1** (1.0 μM) at different time points. The experiment was repeated three times independently, with similar results. The scale bar represents 20 μm.

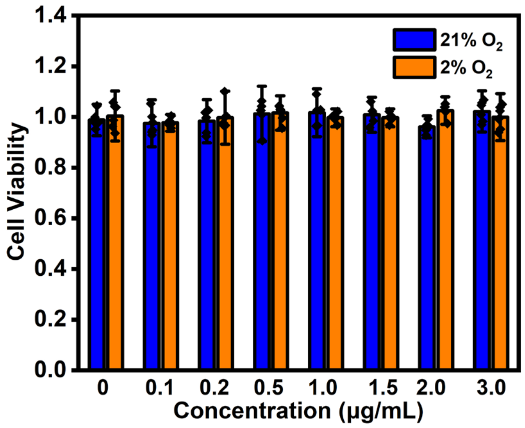

Supplementary Fig. 21 | Cytotoxicity of DA without irradiation. Cell viability of HeLa cells subjected to a range of **DA1** concentrations in the dark under normoxic or hypoxic conditions. Data are presented as mean ± s.d. derived from n = 6 independent biological samples.

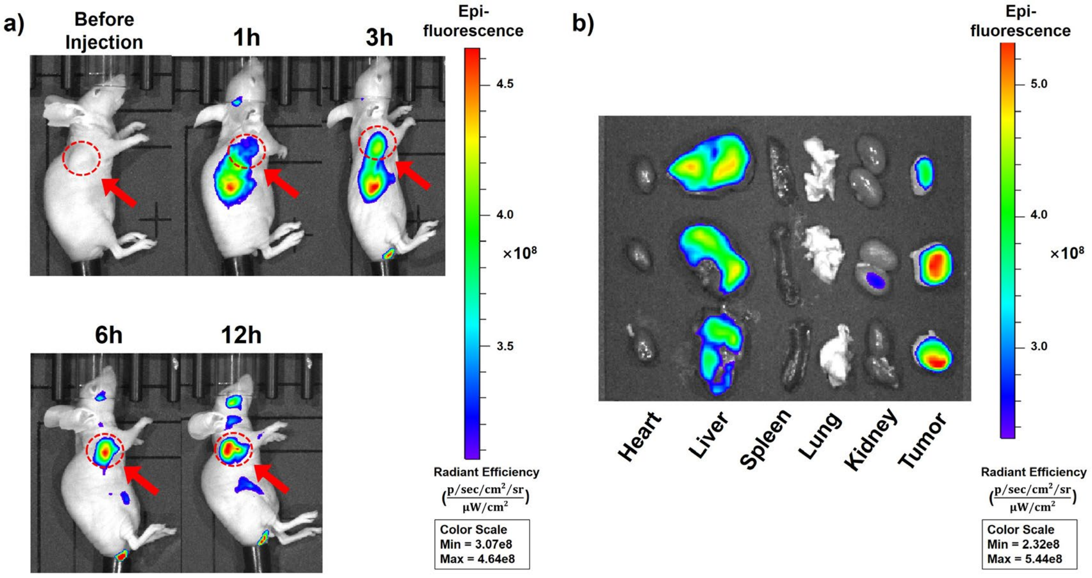

Supplementary Fig. 22 | In vivo fluorescence imaging of DA1. a) In vivo fluorescence imaging of HeLa tumor-bearing BALB/c mice after intravenous injection of **DA1**. b) Fluorescence images of ex vivo organs harvested at 12 hours postinjection.

8. NMR spectra and HRMS

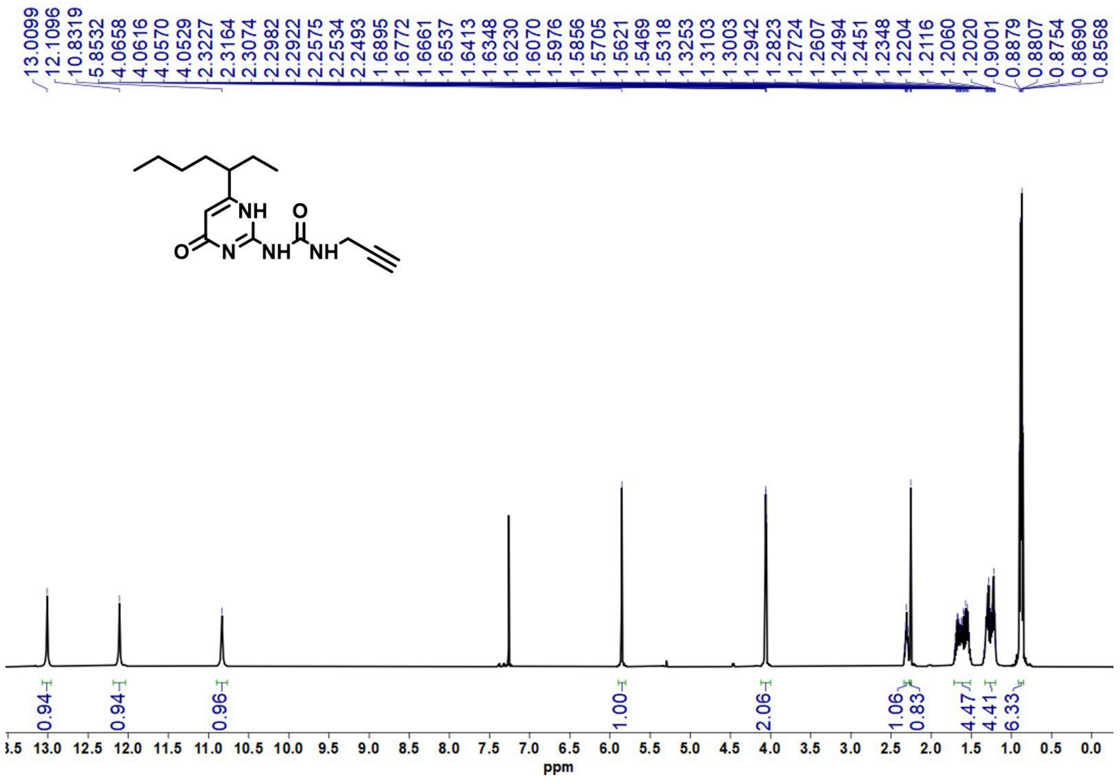

Supplementary Fig. 23 | The ^{1}H NMR of compound 5 in $CDCl_3$.

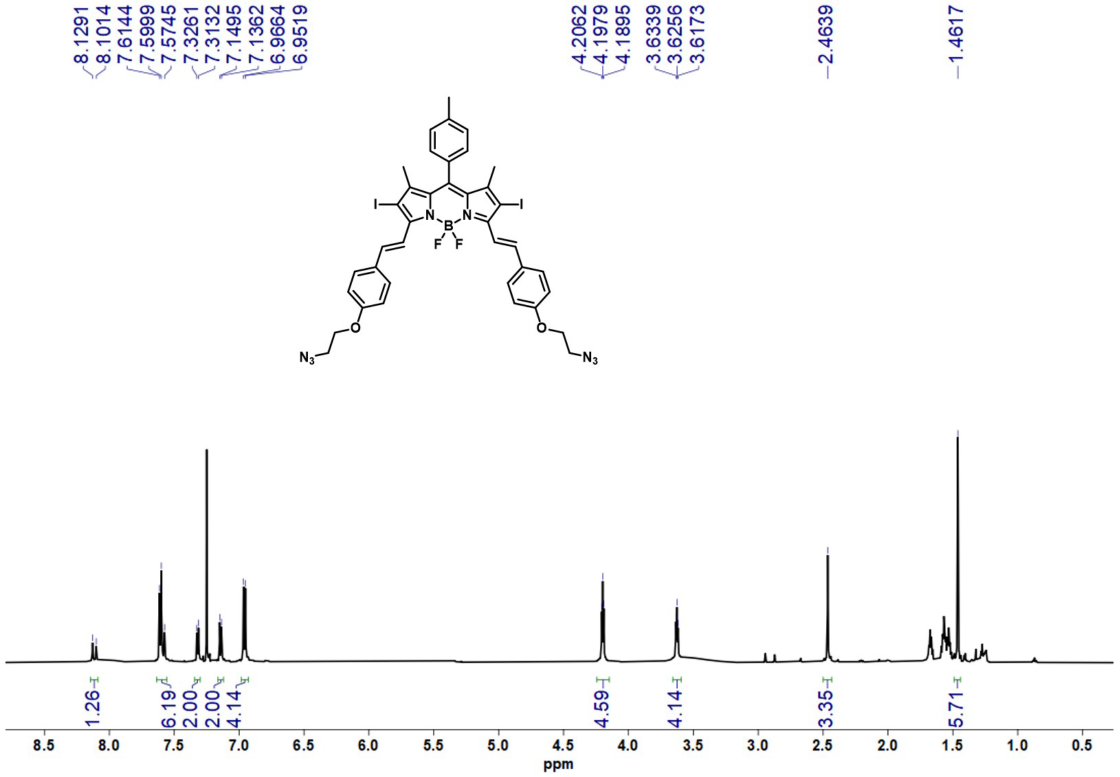

Supplementary Fig. 24 | The ^{1}H NMR of compound 8 in $CDCl_3$.

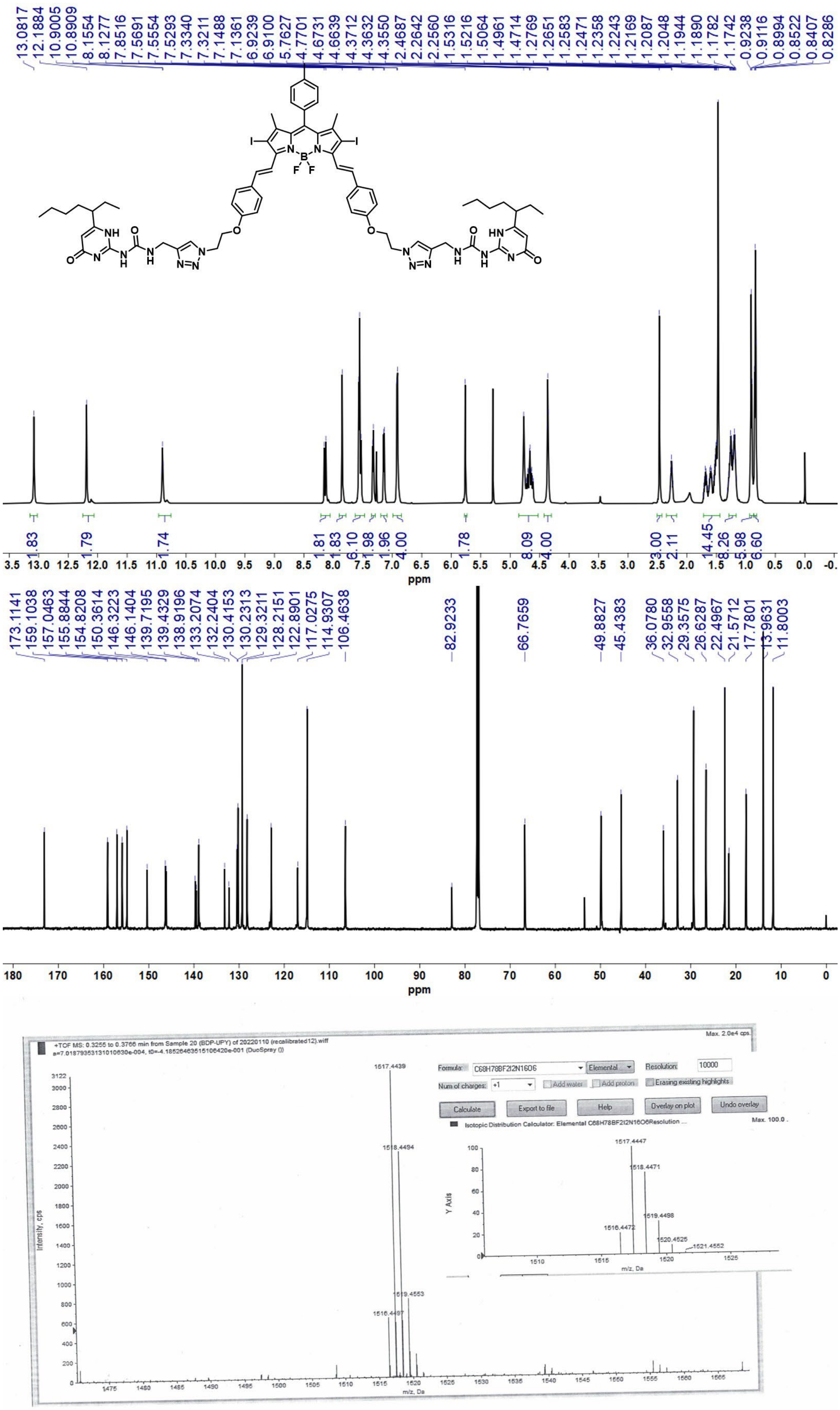

Supplementary Fig. 25 | The ^{1}H NMR ,^{13}C NMR in $CDCl_3$ and HRMS of D.

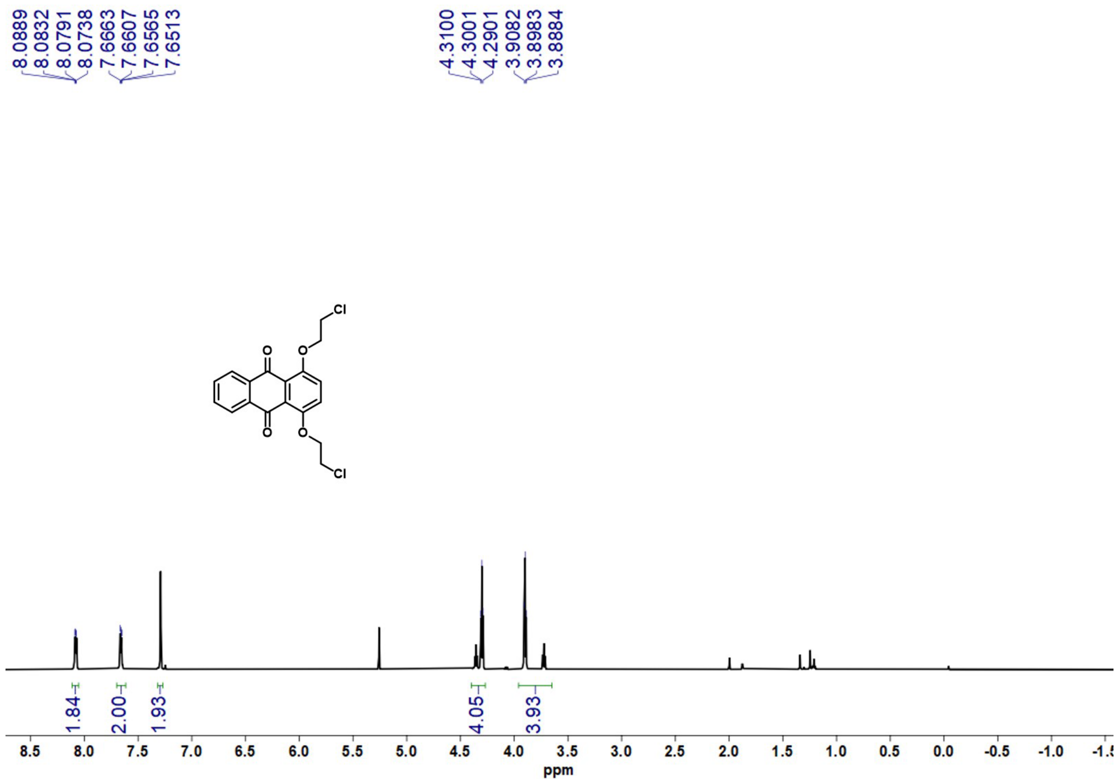

Supplementary Fig. 26 | The ^{1}H NMR of compound 10 in $CDCl_3$.

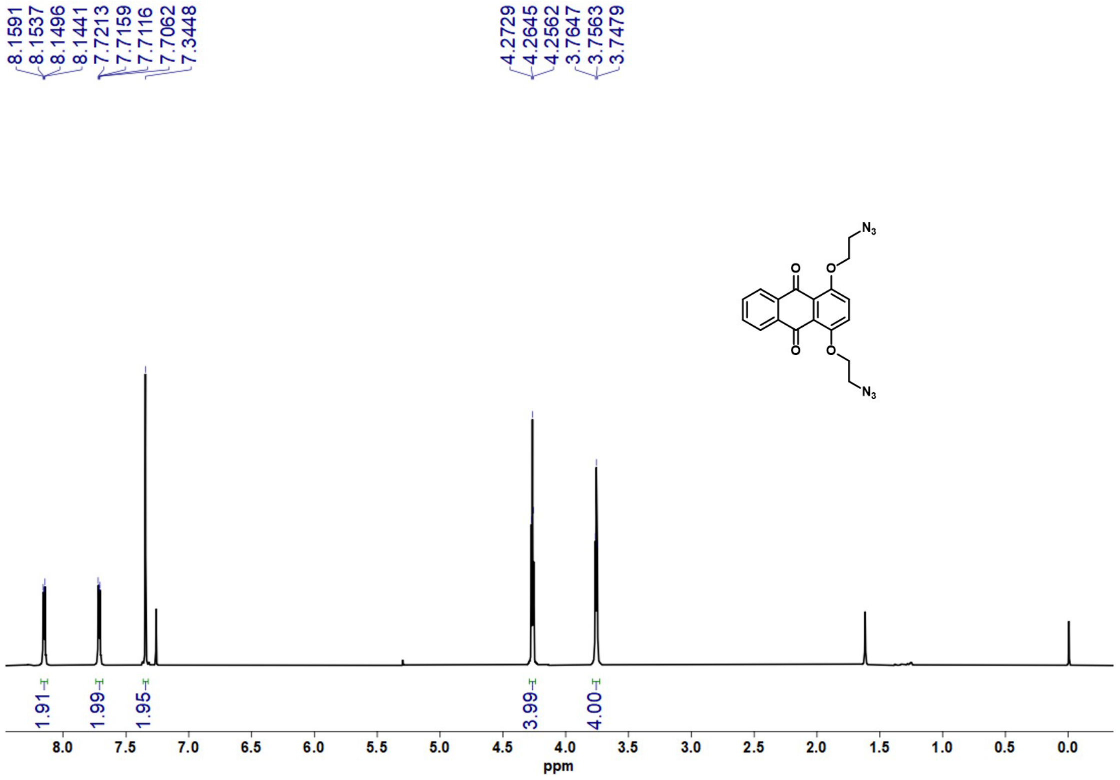

Supplementary Fig. 27 | The ^{1}H NMR of compound 11 in $CDCl_3$.

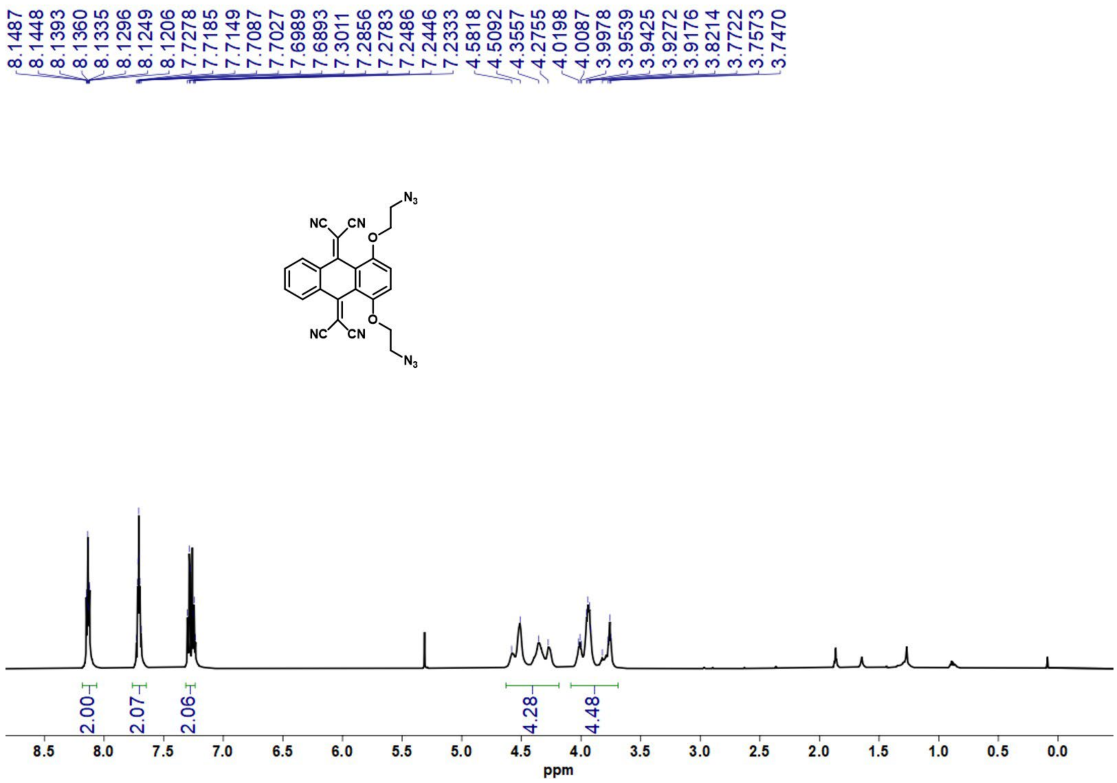

Supplementary Fig. 28 | The ^{1}H NMR of compound 12 in $CDCl_3$.

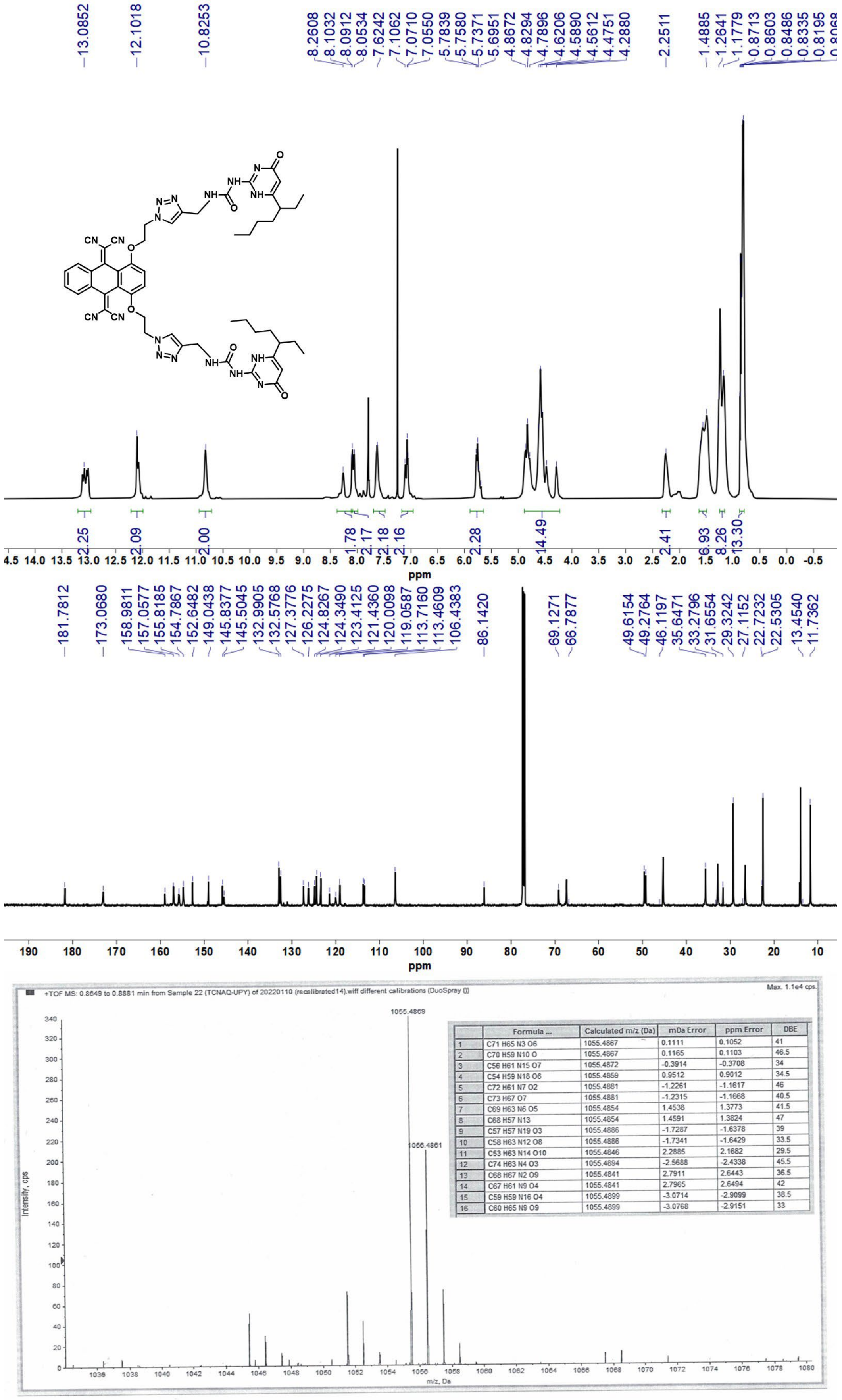

	Formula ...	Calculated m/z (Da)	mDa Error	ppm Error	DBE
1	C71 H65 N3 O6	1055.4867	0.1111	0.1052	41
2	C70 H59 N10 O	1055.4867	0.1165	0.1103	46.5
3	C56 H61 N15 O7	1055.4872	-0.3914	-0.3708	34
4	C54 H59 N18 O6	1055.4859	0.9512	0.9012	34.5
5	C72 H61 N7 O2	1055.4881	-1.2261	-1.1617	46
6	C73 H67 O7	1055.4881	-1.2315	-1.1668	40.5
7	C69 H63 N6 O5	1055.4854	1.4538	1.3773	41.5
8	C68 H57 N13	1055.4854	1.4591	1.3824	47
9	C57 H57 N19 O3	1055.4886	-1.7287	-1.6378	39
10	C58 H63 N12 O8	1055.4886	-1.7341	-1.6429	33.5
11	C53 H63 N14 O10	1055.4846	2.2885	2.1682	29.5
12	C74 H63 N4 O3	1055.4894	-2.5688	-2.4338	45.5
13	C68 H67 N2 O9	1055.4841	2.7911	2.6443	36.5
14	C67 H61 N9 O4	1055.4841	2.7965	2.6494	42
15	C59 H59 N16 O4	1055.4899	-3.0714	-2.9099	38.5
16	C60 H65 N9 O9	1055.4899	-3.0768	-2.9151	33

Supplementary Fig. 29 | The ^{1}H NMR ,^{13}C NMR in $CDCl_3$ and HRMS of A1.

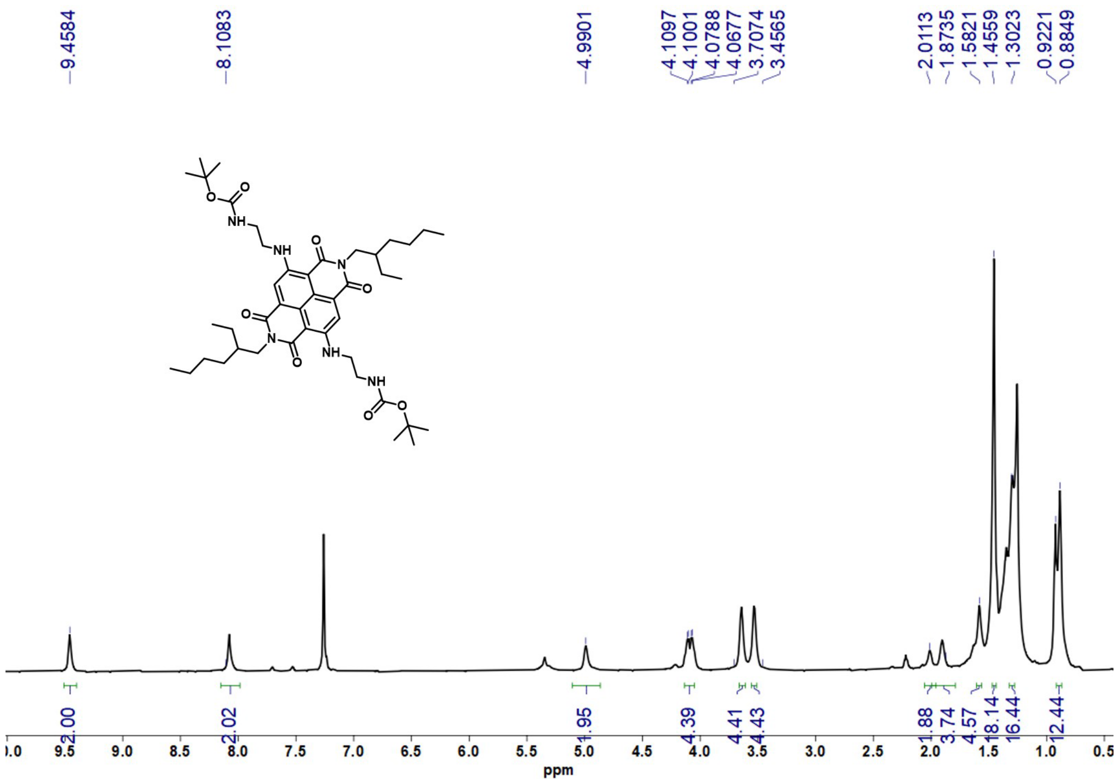

Supplementary Fig. 30 | The ^1H NMR of compound 15 in $CDCl_3$.

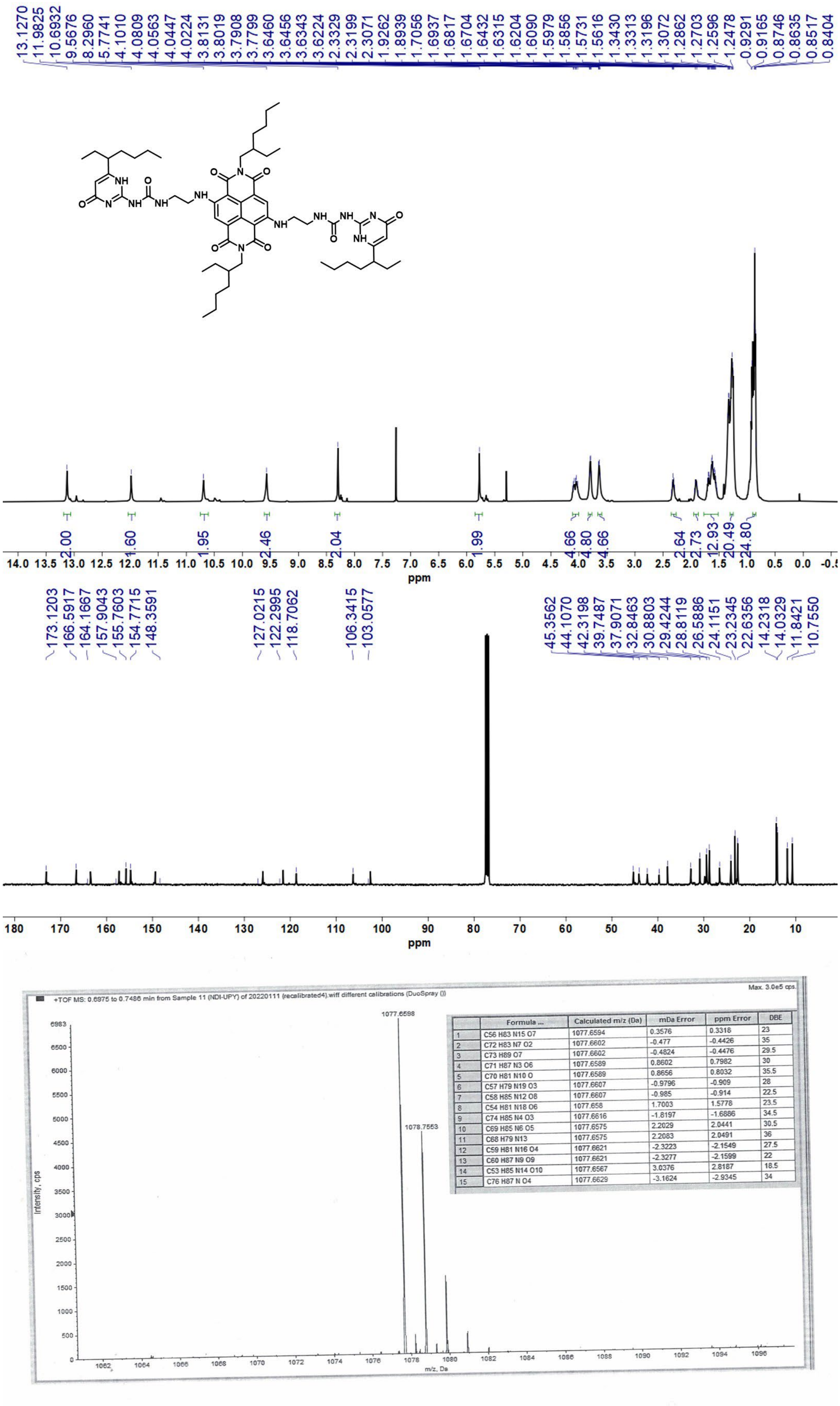

	Formula ...	Calculated m/z (Da)	mDa Error	ppm Error	DBE
1	C56 H83 N15 O7	1077.6594	0.3576	0.3318	23
2	C72 H83 N7 O2	1077.6602	-0.477	-0.4426	35
3	C73 H89 O7	1077.6602	-0.4824	-0.4476	29.5
4	C71 H87 N3 O6	1077.6589	0.8602	0.7982	30
5	C70 H81 N10 O	1077.6589	0.8656	0.8032	35.5
6	C57 H79 N19 O3	1077.6607	-0.9796	-0.909	28
7	C58 H85 N12 O8	1077.6607	-0.985	-0.914	22.5
8	C54 H81 N18 O6	1077.658	1.7003	1.5778	23.5
9	C74 H85 N4 O3	1077.6616	-1.8197	-1.6886	34.5
10	C69 H85 N6 O5	1077.6575	2.2029	2.0441	30.5
11	C68 H79 N13	1077.6575	2.2083	2.0491	36
12	C59 H81 N16 O4	1077.6621	-2.3223	-2.1549	27.5
13	C60 H87 N9 O9	1077.6621	-2.3277	-2.1599	22
14	C53 H85 N14 O10	1077.6567	3.0376	2.8187	18.5
15	C76 H87 N O4	1077.6629	-3.1624	-2.9345	34

Supplementary Fig. 31 | The ^{1}H NMR ,^{13}C NMR in $CDCl_3$ and HRMS of A2.

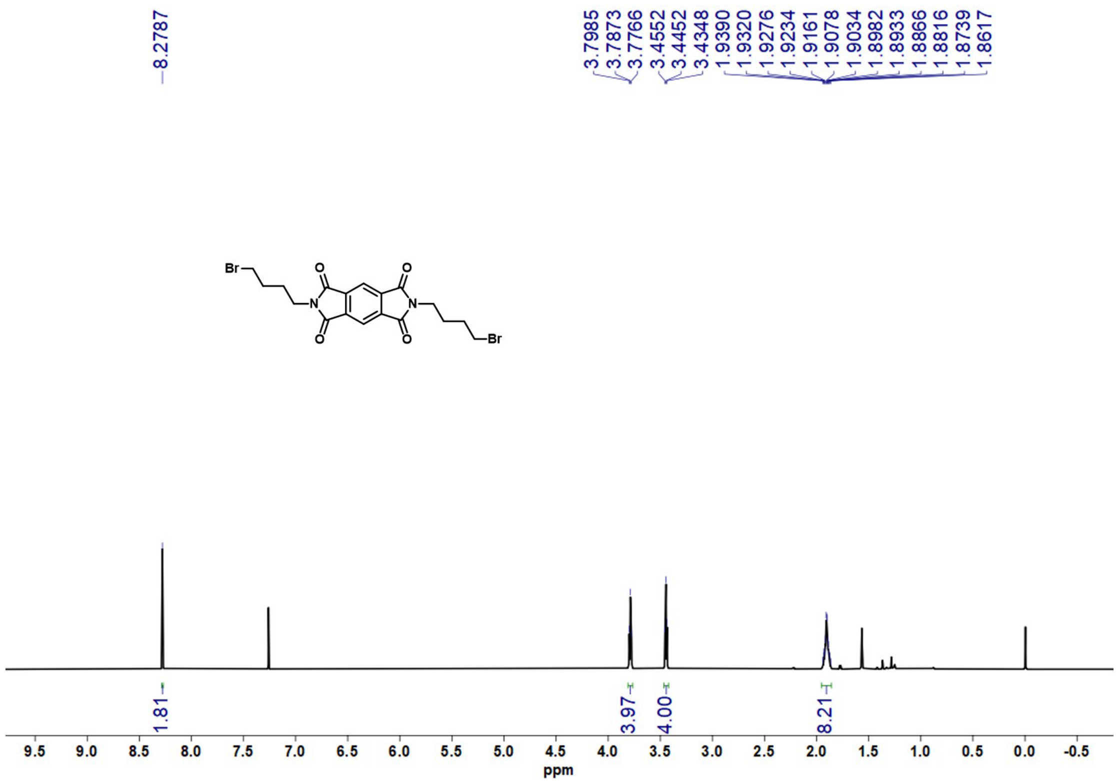

Supplementary Fig. 32 | The ^{1}H NMR of compound 17 in $CDCl_3$.

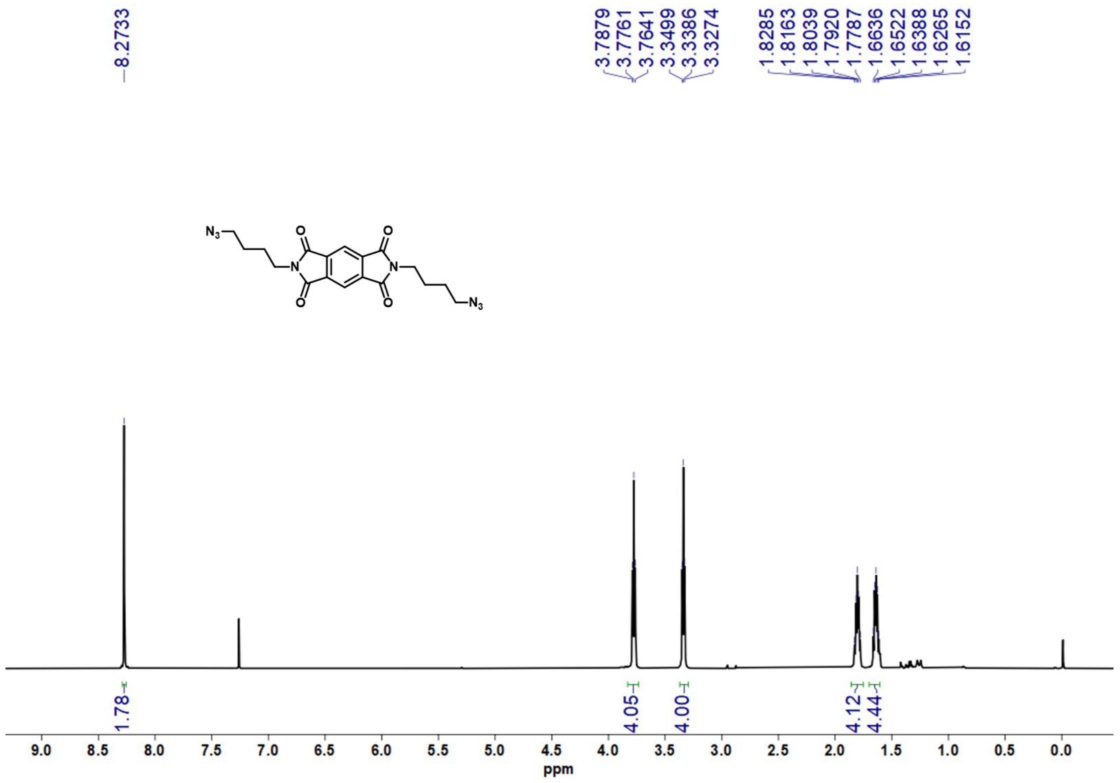

Supplementary Fig. 33 | The ^{1}H NMR of compound 18 in $CDCl_3$.

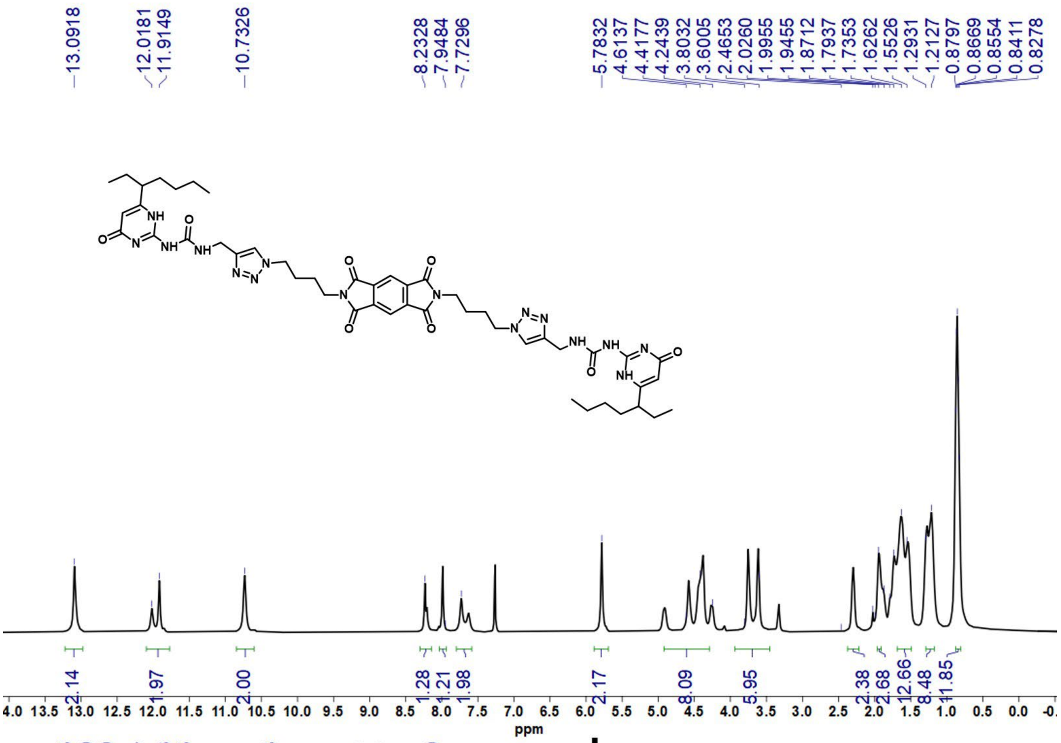

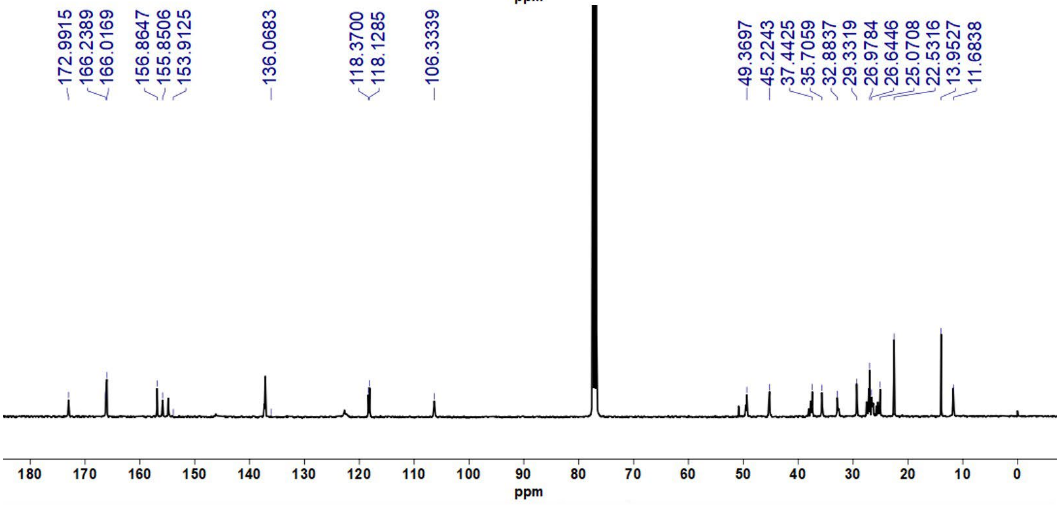

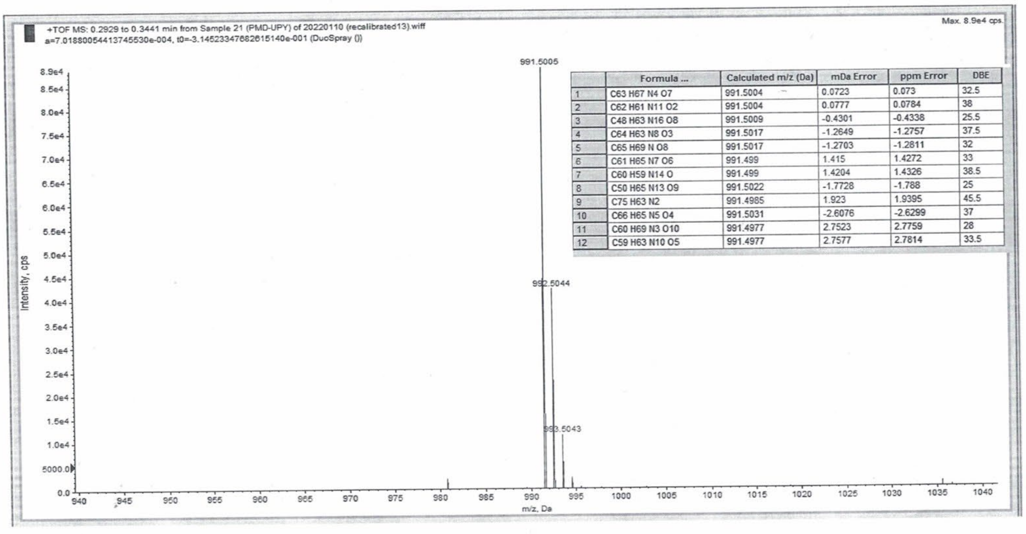

	Formula ...	Calculated m/z (Da)	mDa Error	ppm Error	DBE
1	C63 H67 N4 O7	991.5004	0.0723	0.073	32.5
2	C62 H61 N11 O2	991.5004	0.0777	0.0784	38
3	C48 H63 N16 O8	991.5009	-0.4301	-0.4338	25.5
4	C64 H63 N8 O3	991.5017	-1.2649	-1.2757	37.5
5	C65 H69 N O8	991.5017	-1.2703	-1.2811	32
6	C61 H65 N7 O6	991.499	1.415	1.4272	33
7	C60 H59 N14 O	991.499	1.4204	1.4326	38.5
8	C50 H65 N13 O9	991.5022	-1.7728	-1.788	25
9	C75 H63 N2	991.4985	1.923	1.9395	45.5
10	C66 H65 N5 O4	991.5031	-2.6076	-2.6299	37
11	C60 H69 N3 O10	991.4977	2.7523	2.7759	28
12	C59 H63 N10 O5	991.4977	2.7577	2.7814	33.5

Supplementary Fig. 34 | The ^{1}H NMR , ^{13}C NMR in $CDCl_3$ and HRMS of A3.

9. References

1. Peng, H.-Q.; Chen, Y.-Z.; Zhao, Y.; Yang, Q.-Z.; Wu, L.-Z.; Tung, C.-H.; Zhang, L.-P.; Tong, Q.-X., Artificial light-harvesting system based on multifunctional surface-cross-linked micelles. *Angew. Chem. Int. Ed.* **51**, 2088-2092 (2012).
2. Teng, K.-X.; Niu, L.-Y.; Kang, Y.-F.; Yang, Q.-Z., Rational design of a “dual lock-and-key” supramolecular photosensitizer based on aromatic nucleophilic substitution for specific and enhanced photodynamic therapy. *Chem. Sci.* **11**, 9703-9711 (2020).
3. Li, Z.-J.; Li, S.; Hofman, E.; Hunter Davis, A.; Leem, G.; Zheng, W., Visible-light induced disproportionation of pyrrole derivatives for photocatalyst-free aryl halides reduction. *Green Chem.* **22,** *22*, 1911-1918 (2020).
4. Rehm, D.; Weller, A., Kinetics of fluorescence quenching by electron and H-atom transfer. *Isr. J. Chem.* **8**, 259-271 (1970).
