# Supplementary material for: Supramolecular photodynamic agents for simultaneous oxidation of NADH and generation of superoxide radical
Source: Nat Commun. 2022 Oct 19;13:6179. doi: 10.1038/s41467-022-33924-3 (PMC9582220; doi:10.1038/s41467-022-33924-3)
Supplement: Supplementary file 2 — Reporting Summary [file 41467_2022_33924_MOESM2_ESM.pdf]

nature portfolio

Corresponding author(s): Qing-Zheng Yang
Last updated by author(s): Sep 19, 2022

Reporting Summary

Nature Portfolio wishes to improve the reproducibility of the work that we publish. This form provides structure for consistency and transparency in reporting. For further information on Nature Portfolio policies, see our Editorial Policies and the Editorial Policy Checklist.

Statistics

For all statistical analyses, confirm that the following items are present in the figure legend, table legend, main text, or Methods section.

n/a	Confirmed	
☐	☒	The exact sample size (n) for each experimental group/condition, given as a discrete number and unit of measurement
☐	☒	A statement on whether measurements were taken from distinct samples or whether the same sample was measured repeatedly
☐	☒	The statistical test(s) used AND whether they are one- or two-sided<br>*Only common tests should be described solely by name; describe more complex techniques in the Methods section.*
☒	☐	A description of all covariates tested
☐	☒	A description of any assumptions or corrections, such as tests of normality and adjustment for multiple comparisons
☐	☒	A full description of the statistical parameters including central tendency (e.g. means) or other basic estimates (e.g. regression coefficient) AND variation (e.g. standard deviation) or associated estimates of uncertainty (e.g. confidence intervals)
☐	☒	For null hypothesis testing, the test statistic (e.g. F, t, r) with confidence intervals, effect sizes, degrees of freedom and P value noted<br>*Give P values as exact values whenever suitable.*
☒	☐	For Bayesian analysis, information on the choice of priors and Markov chain Monte Carlo settings
☒	☐	For hierarchical and complex designs, identification of the appropriate level for tests and full reporting of outcomes
☒	☐	Estimates of effect sizes (e.g. Cohen's d, Pearson's r), indicating how they were calculated

Our web collection on statistics for biologists contains articles on many of the points above.

Software and code

Policy information about availability of computer code

Data collection	1H- and 13C-NMR spectra were recorded with JEOL-400, JEOL-600 spectrometers at 298 K. DOSY spectra were recorded with Bruker Avance Drx 500 spectrometer at 298 K. High-resolution mass spectrometry (HRMS) experiments were recorded by an Agilent Technologies 6224 Accurate-Mass time-of-flight spectrometer. Absorption spectra of liquid samples were determined on Hitachi UV-3900 spectrophotometer at room temperature. Fluorescence spectra of liquid samples were determined on Hitachi F-4600 spectrophotometer at room temperature. Cyclic voltammetry was carried out with CHI760E electrochemical workstation. Dynamic light scattering (DLS) investigations were recorded with a DynaPro NanoStar dynamic light scattering detector. Scanning electron microscope (SEM) images were obtained using a Hitachi SU-8010 instrument. The photostability was conducted under irradiation with a high-power LED light and monitored by using an UV-3900 spectrophotometer. Electron spin resonance was performed with Bruker E500. Confocal fluorescence imaging was performed with Nikon A1R microscopy. Cell viability test was obtained on a Thermo Scientific Multiskan. Irradiation was performed by using a LED light (660 nm, PLS-LED 100, Perfect Light, Beijing, China). In vivo imaging was recorded by an IVIS Spectrum imaging system (PerkinElmer, USA).
Data analysis	All ploted and calculated statistical analyses were performed on Origin 8.5 and SPSS.

For manuscripts utilizing custom algorithms or software that are central to the research but not yet described in published literature, software must be made available to editors and reviewers. We strongly encourage code deposition in a community repository (e.g. GitHub). See the Nature Portfolio guidelines for submitting code & software for further information.

Data

Policy information about availability of data

All manuscripts must include a data availability statement. This statement should provide the following information, where applicable:

- Accession codes, unique identifiers, or web links for publicly available datasets
- A description of any restrictions on data availability
- For clinical datasets or third party data, please ensure that the statement adheres to our policy

The data generated in this study are available within the article, Supplementary Information, and Source Data. Source data are provided with this paper.

Human research participants

Policy information about studies involving human research participants and Sex and Gender in Research.

Reporting on sex and gender	N/A
Population characteristics	N/A
Recruitment	N/A
Ethics oversight	N/A

Note that full information on the approval of the study protocol must also be provided in the manuscript.

Field-specific reporting

Please select the one below that is the best fit for your research. If you are not sure, read the appropriate sections before making your selection.

[x] Life sciences [ ] Behavioural & social sciences [ ] Ecological, evolutionary & environmental sciences

For a reference copy of the document with all sections, see nature.com/documents/nr-reporting-summary-flat.pdf

Life sciences study design

All studies must disclose on these points even when the disclosure is negative.

Sample size	Sample size of n ≥ 5 for cell and animal experiments were chosen for statistic analysis.
Data exclusions	No data were excluded.
Replication	Each experiment was repeated at least three times independently and experimental findings were reproducible.
Randomization	The animal used in this paper were randomly distributed into different groups for experiments.
Blinding	The investigators were blinded to group allocation during experiments, data collection and analysis.

Reporting for specific materials, systems and methods

We require information from authors about some types of materials, experimental systems and methods used in many studies. Here, indicate whether each material, system or method listed is relevant to your study. If you are not sure if a list item applies to your research, read the appropriate section before selecting a response.

Materials & experimental systems

n/a	Involved in the study	
x		Antibodies
	x	Eukaryotic cell lines
x		Palaeontology and archaeology
	x	Animals and other organisms
x		Clinical data
x		Dual use research of concern

Methods

n/a	Involved in the study	
x		ChIP-seq
x		Flow cytometry
x		MRI-based neuroimaging

Eukaryotic cell lines

Policy information about cell lines and Sex and Gender in Research

Cell line source(s)	The human cervical cancer cell (HeLa cell) was purchased from The National Experimental Cell Resource Sharing Platform (NICR).
Authentication	These cells were authenticated by cell vitality test, mycoplasma detection and isozyme detection.
Mycoplasma contamination	The cell line tested negative for mycoplasma contamination.
Commonly misidentified lines (See ICLAC register)	No commonly misidentified cell lines are used in this study.

Animals and other research organisms

Policy information about studies involving animals; ARRIVE guidelines recommended for reporting animal research, and Sex and Gender in Research

Laboratory animals	Female BALB/c mice (6-8 weeks) were used as animal model in this study. All mouse models are provided by Beijing Vital River Laboratory Animal Technology Co., Ltd. Mice were housed in individually ventilated cage (IVC) systems (ambient temperature: 23 ± 3 °C; relative humidity: 40-70%).
Wild animals	The study did not involve wild animals.
Reporting on sex	Sex was not considered in the study since our study was not related to sex.
Field-collected samples	The study did not involve samples collected from field.
Ethics oversight	All animal experiments were performed following the protocols evaluated and approved by the Animal Ethics Committee of Capital Medical University (Ethics Approval Number: AEEI-2018-097).

Note that full information on the approval of the study protocol must also be provided in the manuscript.
