# Supplementary figures and images for: Supramolecular photodynamic agents for simultaneous oxidation of NADH and generation of superoxide radical
Source: Nat Commun. 2022 Oct 19;13:6179. doi: 10.1038/s41467-022-33924-3 (PMC9582220; doi:10.1038/s41467-022-33924-3)

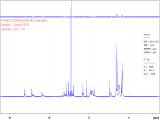

Supplement: Supplementary file 3 — Source Data [file 41467_2022_33924_MOESM3_ESM.zip › Source Data/NMR/dosy/1/1/pdata/1/thumb.png]

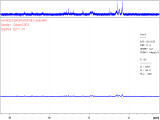

Supplement: Supplementary file 3 — Source Data [file 41467_2022_33924_MOESM3_ESM.zip › Source Data/NMR/dosy/1/2/pdata/1/thumb.png]

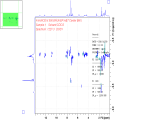

Supplement: Supplementary file 3 — Source Data [file 41467_2022_33924_MOESM3_ESM.zip › Source Data/NMR/dosy/1/3/pdata/1/thumb.png]

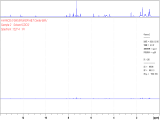

Supplement: Supplementary file 3 — Source Data [file 41467_2022_33924_MOESM3_ESM.zip › Source Data/NMR/dosy/2/4/pdata/1/thumb.png]

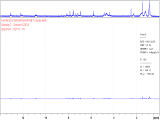

Supplement: Supplementary file 3 — Source Data [file 41467_2022_33924_MOESM3_ESM.zip › Source Data/NMR/dosy/2/5/pdata/1/thumb.png]

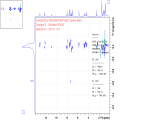

Supplement: Supplementary file 3 — Source Data [file 41467_2022_33924_MOESM3_ESM.zip › Source Data/NMR/dosy/2/6/pdata/1/thumb.png]

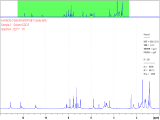

Supplement: Supplementary file 3 — Source Data [file 41467_2022_33924_MOESM3_ESM.zip › Source Data/NMR/dosy/3/7/pdata/1/thumb.png]
